# Per- and Polyfluoroalkyl Substances (PFAS) in Serum of 2 to 5 year-Old Children: Temporal Trends, Determinants, and Correlations with Maternal PFAS Concentrations

**DOI:** 10.1021/acs.est.3c08928

**Published:** 2024-02-09

**Authors:** Jiwon Oh, Hyeong-Moo Shin, Kurunthachalam Kannan, Antonia M. Calafat, Rebecca J. Schmidt, Irva Hertz-Picciotto, Deborah H. Bennett

**Affiliations:** † Department of Public Health Sciences, University of California Davis, Davis, California 95616, United States; ‡ Department of Environmental Science, Baylor University, Waco, Texas 76798, United States; § Division of Environmental Health Sciences, Wadsworth Center, New York State Department of Health, Albany, New York 12201, United States; ∥ Department of Environmental Health Sciences, University at Albany, State University of New York, Albany, New York 12222, United States; ⊥ National Center for Environmental Health, Centers for Disease Control and Prevention, Atlanta, Georgia 30341, United States; # University of California Davis MIND (Medical Investigations of Neurodevelopmental Disorders) Institute, Sacramento, California 98517, United States

**Keywords:** PFAS, child serum, maternal serum, temporal trends, determinants, breastfeeding

## Abstract

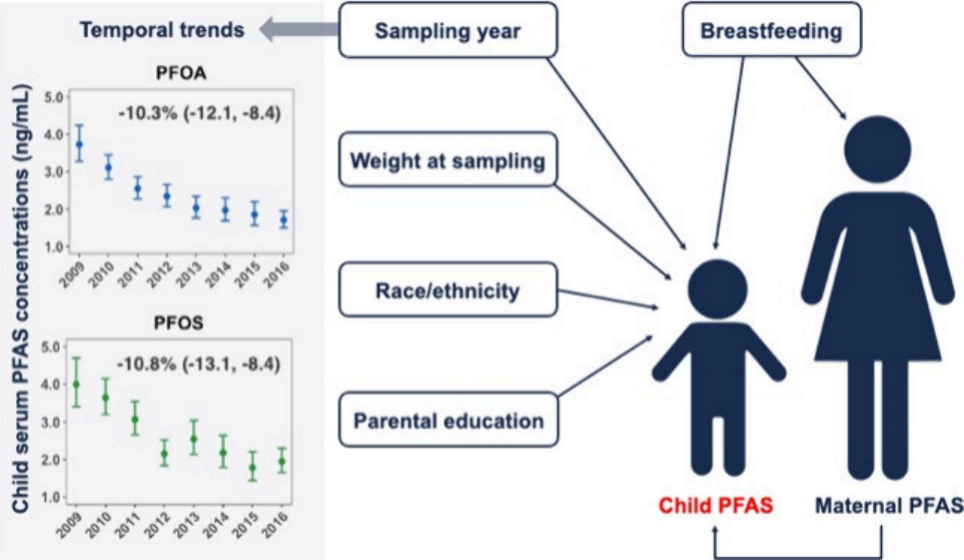

Young children may experience higher per- and polyfluoroalkyl substances (PFAS) exposure than adults due to breastfeeding, higher dust ingestion rates, and frequent hand-to-mouth activities. We explored temporal trends and determinants of child serum PFAS concentrations and their correlations with paired maternal PFAS concentrations. From 2009 to 2017, we collected one blood sample from each of 541 children aged 2–5 years participating in the Childhood Autism Risks from Genetics and Environment (CHARGE) study and quantified 14 PFAS in serum. For nine frequently detected PFAS (>65% of samples), we performed multiple regression adjusting for potential determinants to estimate mean percent concentration changes. For a subset of 327 children, we also quantified nine PFAS in their mother’s serum collected at the same visit and computed Spearman correlation coefficients (*r*_sp_) between maternal and child PFAS concentrations. During 2009–2017, child serum concentrations of all nine PFAS decreased by 6–25% annually. Several PFAS concentrations were higher among non-Hispanic white children and those with highly educated parents. Most maternal and child PFAS concentrations were moderately correlated (*r*_sp_ = 0.13–0.39), with a strong correlation for *N*-methyl perfluorooctane sulfonamido acetic acid (*r*_sp_ = 0.68). Breastfeeding duration appeared to contribute to higher child and lower maternal PFAS concentrations, resulting in relatively weak correlations between maternal and child PFAS concentrations for samples collected in early childhood. Considering that more than half of our study children had neurodevelopmental concerns, the generalizability of our findings might be limited.

## Abstract 2

Childhood body burden of PFAS has decreased from 2009 to 2017; breastfeeding duration is a major determinant.

## 1 Introduction

Per- and polyfluoroalkyl substances (PFAS) are a group of man-made chemicals that have unique surfactant properties as well as chemical and thermal stability.^1^ Since the 1940s, PFAS have been extensively used in nonstick cookware, food packaging products, carpets, furniture, clothing, and fire-fighting foams.^2,3^ Widespread use in industrial and consumer applications and environmental persistence have led to ubiquitous detection of common long-alkyl-chain PFAS in the serum of the United States (U.S.) general population over the past decades.^4^ Several PFAS are reported to have adverse effects on laboratory animals and humans, such as reproductive and developmental toxicity, neurotoxicity, hormone disruption, liver, renal, and cardiovascular toxicity, and immunotoxicity.^5−7^ Due to public health concerns, the 3M Company, one of the major global manufacturers of perfluorooctanesulfonic acid (PFOS) and related compounds, voluntarily discontinued production in 2002.^8,9^ Subsequently, the U.S. Environmental Protection Agency (EPA) and eight leading fluoropolymer and telomer manufacturers agreed to phase out perfluorooctanoic acid (PFOA) and related compounds by 2015.^10^ PFOS and related compounds are listed in Annex B (Restriction) of the Stockholm Convention on Persistent Organic Pollutants, and PFOA, perfluorohexane-1-sulfonic acid (PFHxS), and related compounds are listed in Annex A (Elimination).^11,12^

Children at early ages are exposed to PFAS via various pathways, including breastfeeding, ingestion of contaminated food, water, dust, and soil, and hand-to-mouth contact with indoor surfaces.^13−17^ Because of their relatively high food and dust ingestion rates per body weight and age-specific behaviors, such as frequent hand-to-mouth activity and playing close to the ground, young children may experience higher PFAS exposure than adults.^13,14,18^ Many studies that quantified PFAS in the serum or plasma of children aged 1 to 5 years showed that PFAS were detected in this vulnerable population even after phase-out efforts.^16,17,19−33^ Three studies examining temporal trends of PFAS body burden in children aged 0 to 12 years have consistently observed decreases in concentrations of two major PFAS, PFOS and PFOA, since the early 2000s.^31,34,35^ However, these studies showed mixed temporal trends for PFHxS and perfluorononanoic acid (PFNA) and did not investigate longer- or shorter-alkyl-chain PFAS.

Breastfeeding is an important exposure pathway for several PFAS in breastfed infants,^16,17,36^ contributing to increasing child PFAS concentrations during this life-stage period.^16,29^ Breastfeeding duration is a common determinant of several PFAS concentrations not only in infants and toddlers^17,30,32,33,37^ but also in preschool and school-aged children,^28,29,31^ partly due to the long biological half-lives of many PFAS.^2^ Toddlers and preschoolers, in particular, stop breastfeeding and begin sharing many PFAS exposure sources with their mothers within the home environment by consuming the same water and food and using the same carpets and furniture.^13^ Despite the transition of their exposure sources and partially sharing exposure sources with their mothers, to our knowledge, only one small-scale study investigated correlations between PFAS concentrations in mothers and their young children, which included toddlers and preschoolers, reporting significant but relatively weak to moderate correlations of PFAS concentrations,^28^ while two other studies examined maternal–child correlations in infants^37^ or school-aged children.^38^

This study aimed to investigate temporal trends of PFAS concentrations and the concentration determinants including sociodemographic characteristics and breastfeeding duration. We used serum samples collected from 541 children aged 2–5 years, each providing a single sample between 2009 and 2017. In a subset of 327 mother–child dyads whose serum samples were collected from both mothers and children for PFAS quantification, we examined the correlations between the paired child and maternal serum PFAS concentrations.

## 2 Methods

### 2.1 Study Population and Blood Sample Collection

We used data and biological samples from the Childhood Autism Risks from Genetics and Environment (CHARGE) study, an ongoing case-control study that began full recruitment in 2003.^39^ The CHARGE study recruited children who received services for autism spectrum disorder (ASD) and developmental delay (DD) through Regional Centers funded by the California Department of Health and Human Services (DDS). General population controls were randomly selected from state birth files and frequency-matched to the sex, age, and catchment area of children with ASD. Inclusion criteria were children born in California, 2–5 years of age at enrollment, living with at least one biological parent who speaks English or Spanish, and residing in the catchment areas of a specified list of California Regional Centers in Northern California. If an enrolled child was a twin or triplet, parents had the option of enrolling multiple children in the study. Twins of children with ASD or DD concerns who did not have any neurodevelopmental concerns were defined as unaffected twins. Participating families with other children with an ASD or DD concern who met the other enrollment criteria could enroll those children. Finally, if an enrolled family later had additional children with an ASD or DD concern, they had the option of contacting the study staff and enrolling the additional child.

**Figure 1:**
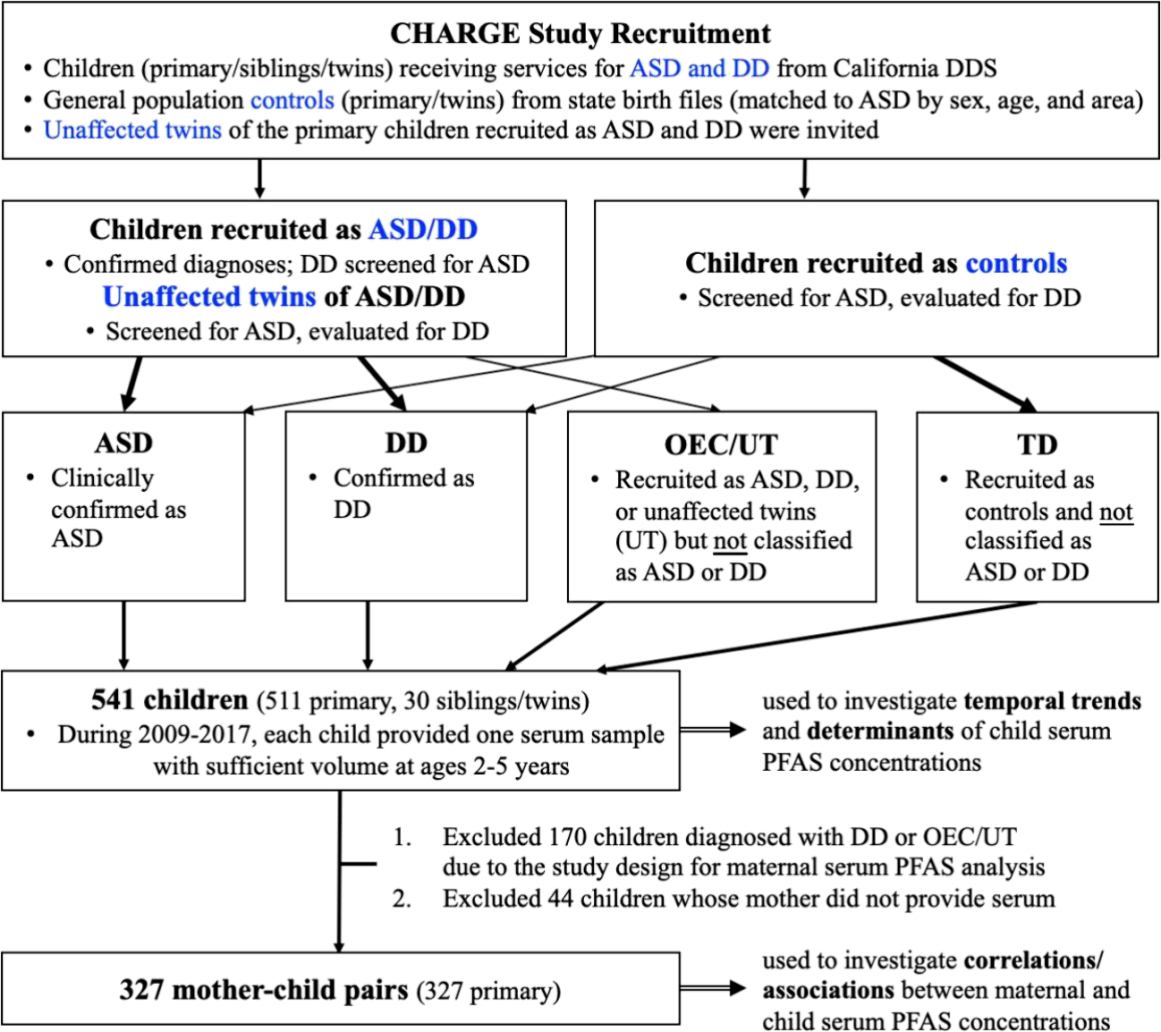
Flowchart describing the recruitment and diagnosis in the CHARGE study and derivation of the child serum sample and the sample of mother–child dyads for this study. ASD, autism spectrum disorder; CHARGE, Childhood Autism Risks from Genetics and Environment; DD, developmental delay; DDS, Department of Developmental Services; OEC/UT, other early concerns/unaffected twin; PFAS, per- and polyfluoroalkyl substances; TD, typical development.

Enrolled children and their mothers visited the University of California Davis Medical Investigations of Neurodevelopmental Disorders (MIND) Institute for neurodevelopmental assessment. Children recruited as ASD were administered a set of standardized clinical assessments to confirm their diagnoses. Children recruited as DD or unaffected twins were screened for ASD and evaluated for DD. Children recruited as ASD, DD, or unaffected twins but were ultimately confirmed to not have ASD or DD in this study were classified into the other early concerns/unaffected twin (OEC/UT) group. Participants who were recruited as controls from the general population were screened for ASD and evaluated for DD, with additional testing as needed, and if they were not classified as ASD or DD, they were classified as typically developing (TD). The flowchart describing the recruitment and diagnosis of the CHARGE study is presented in Figure 1. Further details on study design, subject recruitment, data and sample collection, and diagnostic algorithms are available elsewhere.^39^ This study was approved by the institutional review boards of the State of California and the University of California, and prior to data collection, all participants provided written informed consent. The analysis of coded specimens at the Centers for Disease Control and Prevention (CDC) laboratory was determined by the CDC not to constitute engagement in human subject research.

At the MIND Institute visit, both the child and mother provided a single blood sample. The collected blood sample was processed to separate the serum component, which was then stored at −80 °C until it was shipped to the laboratories. During the period of 2009–2017, a total of 551 child serum samples were collected as part of the CHARGE study, which initiated serum collection in 2009. For this study, we restricted it to 541 children with confirmed diagnoses, comprising 511 primary children and 30 siblings (Figure 1). Maternal serum samples were collected for a subset of primary children diagnosed with ASD or DD, with 327 mothers providing serum samples for PFAS quantification at the same study visit. Therefore, we included 327 mother–child dyads in data analyses using both maternal and child serum samples. The number of child and maternal serum samples collected each year is listed in Table 1.

**Table 1:** Characteristics of the Study Population (541 Children and 327 Mother–Child Dyads)^d^

	**all children** (*n* = 541)^a^		**a subset of mother–child dyads** (*n* = 327)^b^	
**characteristic**	***n***	**%**	***n***	**%**
sampling year				
2009	64	12	33	10
2010	111	21	75	23
2011	84	16	44	13
2012	73	13	47	14
2013	54	10	31	9
2014	46	9	31	9
2015	40	7	33	10
2016	50	9	31	9
2017	19	4	2	1
child sex				
female	141	26	61	19
male	400	74	256	81
child age (years) at sampling				
2	86	16	55	17
3	176	33	105	32
4	270	50	164	50
5	9	2	3	1
birth type				
singleton	500	92	310	95
multiple	41	8	17	5
child race/ethnicity				
non-Hispanic white	242	45	158	49
Hispanic	177	33	91	28
Black/Asian/multiracial	114	21	75	23
diagnostic group				
TD	181	33	174	53
ASD	190	35	153	47
DD	103	19	0	0
OEC/UT^c^	67	12	0	0
highest parental education				
less than 4 year college degree	265	49	144	44
bachelor’s degree	176	33	123	38
graduate or professional degree	100	18	60	18
homeownership				
non-owner	220	42	119	38
owner	298	58	196	62
ever breastfed				
no	36	7	18	6
yes	486	93	298	94

	mean ± SD	range	mean ± SD	range
child body weight at sampling (kg)	17 ± 3	10–38	17 ± 3	11–38
maternal age at delivery (years)	30 ± 6	16–47	30 ± 6	16–47
breastfeeding duration (months)	9 ± 9	0–52	9 ± 9	0–52

a Missing (frequency): child race/ethnicity (8), homeownership (24), ever breastfed (19), child body weight at sampling (4), and breastfeeding duration (35).

b Missing (frequency): child race/ethnicity (3), homeownership (12), ever breastfed (11), child body weight at sampling (2), and breastfeeding duration (17).

c OEC/UT groups included 57 children with other early concerns and 10 unaffected twins.

d Note: ASD, autism spectrum disorder; DD, developmental delay; OEC/UT, other early concerns/unaffected twin; SD, standard deviation; TD, typical development.

### 2.2 Serum PFAS Quantification

Fourteen PFAS were quantified in child serum at Wadsworth Center’s Human Health Exposure Analysis Resource Targeted Analysis Laboratory using hybrid-solid-phase extraction (SPE) and high-performance liquid chromatography (HPLC) coupled with electrospray triple-quadrupole tandem mass spectrometry, as published elsewhere.^40,41^ The quantified PFAS included 8 perfluoroalkyl carboxylic acids (PFCAs) [i.e., perfluoro-*n*-pentanoic acid (PFPeA), perfluorohexanoic acid (PFHxA), perfluoroheptanoic acid (PFHpA), PFOA, PFNA, perfluorodecanoic acid (PFDA), perfluoroundecanoic acid (PFUnDA), and perfluorododecanoic acid (PFDoDA)], 3 perfluoroalkanesulfonic acids (PFSAs) [i.e., perfluorobutanesulfonic acid (PFBS), PFHxS, and PFOS], and 3 perfluoroalkane sulfonamides [i.e., perfluorooctanesulfonamide (FOSA), *N*-methyl perfluorooctane sulfonamido acetic acid (MeFOSAA), and *N*-ethyl perfluorooctane sulfonamido acetic acid (EtFOSAA)]. The study samples were analyzed with procedural blanks, quality control samples spiked with standards at 5 ng for all analytes and internal standards (5 ng/mL serum), and replicates of Standard Reference Materials (SRM1957 and SRM1958, NIST, Gaithersburg, MD, USA). For quality assurance, 19 blinded duplicate samples were also analyzed. The median relative percentage differences of duplicates when PFAS were above the limit of detection (LOD) in both duplicates ranged from 5 to 20% depending on the analyte. The LODs in child serum were 0.05 ng/mL for PFPeA, PFHxA, PFOA, and PFDoDA and 0.02 ng/mL for PFHpA, PFNA, PFDA, PFUnDA, PFBS, PFHxS, PFOS, FOSA, MeFOSAA, and EtFOSAA.

Nine PFAS (PFHxS, PFOS, PFOA, PFNA, PFDA, PFUnDA, PFDoDA, MeFOSAA, and EtFOSAA) were quantified in maternal serum at the CDC using online SPE and reversed-phase HPLC-isotope dilution tandem mass spectrometry, as described elsewhere.^42^ Procedural blanks and quality control serum samples spiked with low and high concentrations of the target analytes were included in each batch, along with analytical standards. The median coefficient of variation of 25 blinded duplicates analyzed with the study samples ranged from 0 to 11% depending on PFAS. The LOD in maternal serum was 0.1 ng/mL for all nine PFAS. Despite different SPE methods used by the two laboratories for sample extraction, matrix spike recoveries between the methods were comparable (89–117% for child samples and 87–108% for maternal samples).^40,42^ Both laboratories regularly and successfully participate in external quality assessment schemes, which ensures the comparability of the different analytical methods.

### 2.3 Determinants of PFAS Concentrations

Sociodemographic characteristics of mother–child dyads were obtained from questionnaires, and potential determinants of child serum PFAS concentrations were identified *a priori* based on the review of information found in the literature. These potential determinants included: sampling year (year), child age (month) and weight (kg) at sampling, maternal age at delivery (year), and breastfeeding duration (month). Child race/ethnicity (non-Hispanic white, Hispanic, and Black/Asian/multiracial), highest parental education (less than 4 year college degree, bachelor’s degree, and graduate or professional), and homeownership (non-owner and owner) were also considered as proxies for socioeconomic status. Because there were only 19 child serum samples collected in 2017, we combined samples collected in 2016 and 2017 (*n* = 69) for statistical analysis. We also considered child diagnosis of ASD, DD, OEC/UT, and TD as a potential determinant as our prior study found significant cross-sectional associations between these diagnoses and child PFAS concentrations in the CHARGE study.^41^

### 2.4 Statistical Analysis

For statistical analysis of child PFAS concentrations in 541 study children, we included nine frequently detected (>65%) PFAS (i.e., PFPeA, PFHpA, PFOA, PFNA, PFDA, PFUnDA, PFHxS, PFOS, and MeFOSAA), while other PFAS were excluded due to low detection frequency (<30%).^43^ Among them, five PFAS (i.e., PFHxS, PFOS, PFOA, PFNA, and PFDA) frequently detected (>65%) in the maternal serum samples were included for data analysis in 327 mother–child pairs while excluding the other four PFAS due to the relatively low detection frequency (<50%). We substituted PFAS concentrations below the LOD with a value of LOD divided by the square root of 2. We used the Wilcoxon rank-sum test to compare the five PFAS concentrations between child and maternal serum samples in the subset of 327 mother–child dyads. We calculated the Spearman correlation coefficients (*r*_sp_) between child and maternal serum concentrations of five PFAS only when both maternal and child PFAS were detectable.

In 541 children, we examined bivariate associations between 9 child serum PFAS concentrations and each potential determinant using the Spearman correlation test for continuous variables, the Wilcoxon rank-sum test for binary variables, and the Kruskal–Wallis test for categorical variables with >2 levels. We selected potential determinants that were associated with ≥3 PFAS concentrations (*p* < 0.05) as covariates for the multivariate analyses: child age and body weight at sampling, breastfeeding duration, child race/ethnicity, diagnostic group, and highest parental education. To impute missing covariates, we performed multiple imputation with chained equations, which included all PFAS concentrations and potential determinants, generating 20 imputed data sets.^44,45^

We performed linear regression with natural log (ln)-transformed child serum PFAS concentrations as the dependent variables, sampling year (centered to 2012) as a primary independent variable, and selected covariates. Although there was a moderate correlation between child age and body weight at sampling, the generalized variance inflation factors in the regression models were low (range = 1.03–1.26), indicating minimal multicollinearity between the independent variables.^46^ Therefore, we decided to retain both variables in the models. First, we calculated percent changes in child serum PFAS concentrations per sampling year and unit increase in each covariate using the formula (exp(β) – 1) × 100, where β is the regression coefficient for each independent variable, with 95% confidence intervals (CIs) as (exp(β ± 1.96 × SE_β_) – 1) × 100, where SE_β_ is the standard error of the regression coefficients for each independent variable.^47^ The standard errors were adjusted using clustered sandwich variance estimators to account for within-family correlations, as this study population included multiple children born from the same mother. Second, we estimated the least-squares means (LSMs), which are the year-specific means of ln-transformed PFAS concentrations after adjusting for covariates, using an R package “emmeans”.^48^ We then calculated the least-squares geometric means (LSGMs) of PFAS concentrations for each sampling year by exponentiating the LSMs, with 95% CIs as LSM ± 1.96 × SE_LSM_, where SE_LSM_ is the standard error of the LSM adjusted using clustered sandwich variance estimators. We also estimated pooled coefficients of determination (*R*^2^) of the regression models fitted to 20 imputed data sets to assess the percentage of variation in child PFAS concentrations explained by the regression models.

To investigate how much of the variation in child serum PFAS concentrations was explained by maternal serum concentrations of corresponding PFAS, we reran the regression models for 327 mother–child dyads as a sensitivity analysis and then additionally adjusted for ln-transformed maternal serum concentrations in corresponding regression models for five PFAS. We evaluated *R*^2^ values for the regression models before and after adjusting for maternal PFAS concentrations.

We used R version 4.1.3 (R Foundation for Statistical Computing, Vienna, Austria) for all statistical analyses.

## 3 Results

### 3.1 Participant Characteristics

The majority of the 541 child serum samples (62%) were collected in the earlier study period (2009–2012) compared to those in the later study period (2013–2017) (Table 1). Approximately 74% of the children were males, aligning with the CHARGE study’s goal to match the frequencies of child sex between ASD and TD groups due to the higher prevalence of males within the ASD population. The children’s average age at sampling was 46 months (range: 24–60 months), and the average body weight was 17.0 kg (range: 10–38 kg). Approximately 93% of children were ever breastfed, and the average breastfeeding duration was 8.5 months (range: 0–52 months). The sample consisted of 45% non-Hispanic white, 33% Hispanic, and 21% other race (4% non-Hispanic black, 4% Asian, and 13% multiracial) children. More than half of the children were clinically diagnosed with ASD or DD at sampling. Nearly half of children were from families whose highest education level was less than 4 year college degree, and 58% were from families who owned a home. The participant characteristics of the 327 mother–child pairs were generally similar, except that this subset consisted of ASD and TD children only.

### 3.2 PFAS Concentrations in Child and Maternal Serum Samples

Among PFAS quantified in 541 child serum samples, PFPeA, PFHpA, PFOA, PFNA, PFDA, PFHxS, and PFOS were detected in >95% of the samples; PFUnDA and MeFOSAA were detected in 74 and 81% of the samples, respectively (Table 2). The other five PFAS were detected in <30% of samples. The highest median concentration was observed for PFOS (2.45 ng/mL), followed by PFOA (2.38 ng/mL), PFNA (0.85 ng/mL), PFHxS (0.64 ng/mL), and PFPeA (0.57 ng/mL). Among the nine PFAS detected in >65% of the child serum samples, all but PFPeA were positively and weakly to moderately correlated with each other (*r*_sp_ = 0.15–0.68) (Figure S1). In the subset of 327 mother–child dyads, PFOA, PFNA, and PFHxS concentrations were higher in child samples than maternal samples, while PFOS concentrations were higher in maternal samples (Table S1 and Figure S2). When using PFAS detected in both maternal and child samples, PFOA, PFNA, PFDA, PFUnDA, PFHxS, and PFOS showed weak to moderate positive correlations between maternal and child serum samples (*r*_sp_ = 0.13, 0.31, 0.39, 0.38, 0.36, and 0.26 respectively), while MeFOSAA exhibited the strongest positive correlation (*r*_sp_ = 0.68) (Table S2). Child serum concentrations of nine PFAS differed across several participant characteristics, including sampling year, child age and body weight at sampling, race/ethnicity, diagnostic group, highest parental education, homeownership, and breastfeeding duration (Table S3).

**Table 2:** Distribution of Serum PFAS Concentrations in 541 CHARGE Children^a^

				**percentile (ng/mL)**				
**class/group**	**PFAS**	**LOD** (ng/mL)	**% >LOD**	**5th**	**25th**	**50th**	**75th**	**95th**
PFCAs	PFPeA	0.05	99.4	0.22	0.38	0.57	0.79	1.25
	PFHxA	0.05	16.1	<LOD	<LOD	<LOD	<LOD	0.28
	PFHpA	0.02	96.5	0.03	0.11	0.21	0.37	0.87
	PFOA	0.05	100.0	0.96	1.65	2.38	3.68	6.81
	PFNA	0.02	100.0	0.29	0.56	0.85	1.24	2.60
	PFDA	0.02	99.6	0.07	0.11	0.16	0.24	0.48
	PFUnDA	0.02	74.3	<LOD	<LOD	0.04	0.07	0.15
	PFDoDA	0.05	4.1	<LOD	<LOD	<LOD	<LOD	<LOD
PFSAs	PFBS	0.02	29.8	<LOD	<LOD	<LOD	0.02	0.07
	PFHxS	0.02	100.0	0.24	0.38	0.64	1.15	3.39
	PFOS	0.02	100.0	0.93	1.60	2.45	4.07	11.00
perfluoroalkane sulfonamides	FOSA	0.02	0.7	<LOD	<LOD	<LOD	<LOD	<LOD
	MeFOSAA	0.02	81.1	<LOD	0.04	0.12	0.36	1.64
	EtFOSAA	0.02	17.0	<LOD	<LOD	<LOD	<LOD	0.07

a Note: CHARGE, Childhood Autism Risks from Genetics and Environment; FOSA, perfluorooctanesulfonamide; EtFOSAA, *N*-ethyl perfluorooctane sulfonamido acetic acid; LOD, limit of detection; MeFOSAA, *N*-methyl perfluorooctane sulfonamido acetic acid; PFAS, per- and polyfluoroalkyl substances; PFBS, perfluorobutanesulfonic acid; PFCA, perfluoroalkyl carboxylic acids; PFDA, perfluorodecanoic acid; PFHpA, perfluoroheptanoic acid; PFDoDA, perfluorododecanoic acid; PFHxA, perfluorohexanoic acid; PFHxS, perfluorohexane-1-sulfonic acid; PFNA, perfluorononanoic acid; PFOA, perfluorooctanoic acid; PFOS, perfluorooctanesulfonic acid; PFPeA, perfluoro-*n*-pentanoic acid; PFSA, perfluoroalkanesulfonic acids; PFUnDA, perfluoroundecanoic acid.

### 3.3 Temporal Trends and Determinants of Child Serum PFAS Concentrations

**Figure 2:**
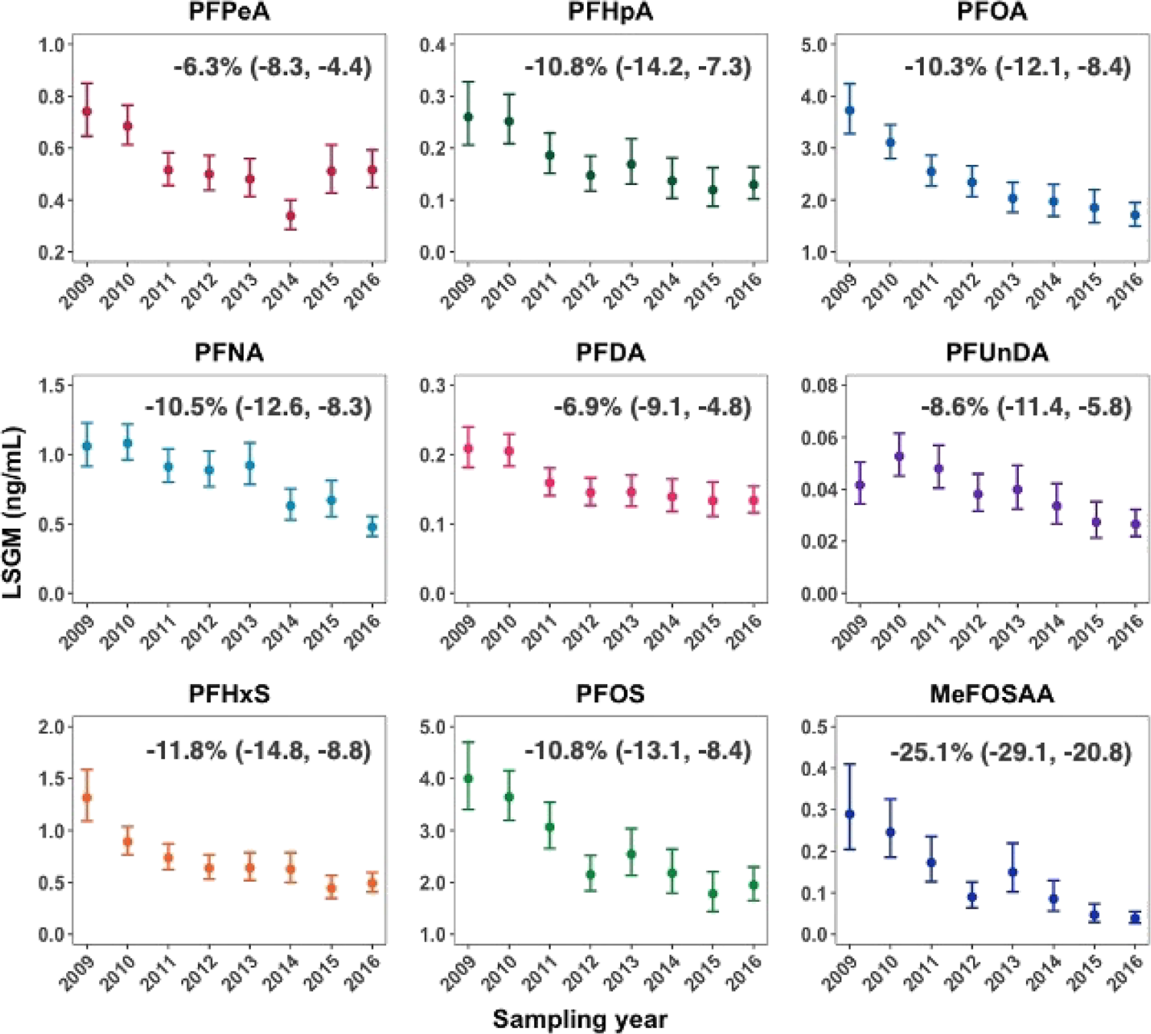
Temporal trends of child serum PFAS concentrations during 2009–2017 in 541 CHARGE children. The point estimates represent LSGMs, and error bars represent their corresponding 95% CIs. The percentages presented on the top right corner represent adjusted annual mean percent changes (95% CIs). The serum samples for 2016 and 2017 were combined due to the small number of samples collected in 2017. Linear regression models were adjusted for child age and body weight at sampling, breastfeeding duration, child race/ethnicity, diagnostic group, and highest parental education. CHARGE, Childhood Autism Risks from Genetics and Environment; CI, confidence interval; LSGM, least-squares geometric mean; MeFOSAA, *N*-methyl perfluorooctane sulfonamido acetic acid; PFAS, per- and polyfluoroalkyl substances; PFDA, perfluorodecanoic acid; PFHpA, perfluoroheptanoic acid; PFHxS, perfluorohexane-1-sulfonic acid; PFNA, perfluorononanoic acid; PFOA, perfluorooctanoic acid; PFOS, perfluorooctanesulfonic acid; PFPeA, perfluoro-*n*-pentanoic acid; PFUnDA, perfluoroundecanoic acid.

From 2009 to 2017, LSGM concentrations of all nine PFAS in 541 child serum samples declined by 6 to 25% per year (Figure 2). The greatest decrease was observed for MeFOSAA (percent change per year: −25.1%), followed by PFHxS (−11.8%), PFHpA (−10.8%), PFOS (−10.8%), PFNA (−10.5%), and PFOA (−10.3%). LSGM concentrations of PFPeA, PFDA, and PFUnDA also decreased but to a lesser extent (−6.3, −6.9, and −8.6%, respectively). Most of these PFAS, except PFNA and PFUnDA, showed curvilinear relationships, with the sharpest declines during 2009–2011 and then leveling off during 2011–2017. On the contrary, PFNA and PFUnDA gradually decreased during 2009–2011 and then more rapidly decreased during 2011–2017.

Several participant characteristics were associated with child serum PFAS concentrations (Table 3). Higher children’s age at sample collection was associated with higher PFPeA concentrations (1% per month) and with lower PFHpA concentrations (−2% per month). Higher children’s body weight at sampling was associated with lower concentrations of PFOA and PFDA (−2% per kg). Longer breastfeeding duration was associated with higher concentrations of seven PFAS (1–3% per month, which is as high as 17% per 6 months), except PFPeA and MeFOSAA. Black, Asian, or multiracial children had 14% to 23% lower concentrations of PFHpA, PFOA, and PFNA, compared with non-Hispanic white children. Children with ASD had higher concentrations of PFHpA (38%) and PFOA (16%) and lower PFUnDA concentrations (−27%) compared to TD children. Children in the OEC/UT group had 21 to 27% lower concentrations of PFDA and PFUnDA than those with TD. Compared with children whose parents’ highest education attainment was less than 4 year college degree, children who had at least one parent with a graduate or professional degree had 21% higher PFDA and 29% higher PFOS concentrations.

**Table 3:** Adjusted Mean Percent Changes (95% CIs) in Serum PFAS Concentrations per One-Unit Increase of Each Potential Determinant in 541 CHARGE Children^b^

potential determinant	PFPeA	PFHpA	PFOA	PFNA	PFDA	PFUnDA	PFHxS	PFOS	MeFOSAA
sampling year (year)	****–**6.3 (−8.3, −4.4)**	****–**10.8 (−14.2, −7.3)**	****–**10.3 (−12.1, −8.4)**	****–**10.5 (−12.6, −8.3)**	****–**6.9 (−9.1, −4.8)**	****–**8.6 (−11.4, −5.8)**	****–**11.8 (−14.8, −8.8)**	****–**10.8 (−13.1, −8.4)**	****–**25.1 (−29.1, −20.8)**
child age at sampling (month)	**0.9 (0.2, 1.5)**	****–**2.0 (−3.1, −0.8)**	0.0 (−0.6, 0.6)	0.5 (−0.2, 1.3)	0.4 (−0.3, 1.1)	1.0 (−0.1, 2.0)	0.0 (−0.8, 0.9)	0.0 (−0.8, 0.8)	–0.6 (−2.2, 1.0)
child body weight at sampling (kg)	–0.3 (−2.1, 1.5)	–0.7 (−3.5, 2.1)	****–**1.6 (−3.2, 0.0)**	–1.3 (−3.0, 0.5)	****–**1.8 (−3.4, −0.3)**	–1.7 (−4.1, 0.7)	–1.1 (−3.8, 1.7)	–2.0 (−4.0, 0.1)	–2.0 (−6.1, 2.3)
breastfeeding duration (month)	–0.6 (−1.7, 0.5)	**1.8 (0.9, 2.7)**	**2.8 (2.2, 3.4)**	**1.7 (1.1, 2.3)**	**1.5 (0.8, 2.3)**	**1.2 (0.3, 2.1)**	**2.5 (1.7, 3.3)**	**2.7 (1.9, 3.5)**	–0.4 (−1.8, 1.0)
									
child race/ethnicity									
non-Hispanic white	ref	ref	ref	ref	ref	ref	ref	ref	ref
Hispanic	5.1 (−6.3, 17.9)	–6.4 (−23.1, 13.9)	–9.6 (−18.6, 0.3)	8.1 (−4.2, 22.1)	–1.1 (−11.3, 10.4)	12.4 (−3.8, 31.2)	–8.6 (−22.4, 7.6)	–5.1 (−17.4, 9.2)	–17.5 (−38.9, 11.4)
Black/Asian/multiracial	–2.2 (−14.5, 11.7)	****–**22.2 (−37.9, −2.6)**	****–**13.9 (−24.0, −2.6)**	****–**15.3 (−25.8, −3.5)**	–12.3 (−24.1, 1.4)	0.7 (−16.0, 20.7)	–10.7 (−24.6, 5.8)	–13.2 (−25.3, 1.0)	–6.4 (−32.8, 30.3)
									
diagnostic group									
TD	ref	ref	ref	ref	ref	ref	ref	ref	ref
ASD	6.2 (−6.9, 21.1)	**37.5 (12.7, 67.8)**	**15.6 (3.9, 28.7)**	–9.6 (−20.7, 3.1)	–6.1 (−17.3, 6.7)	****–**26.6 (−38.1, −12.9)**	8.3 (−7.3, 26.5)	–1.6 (−14.0, 12.5)	8.3 (−19.6, 45.8)
DD	10.9 (−3.6, 27.6)	–15.2 (−33.7, 8.4)	–1.0 (−14.0, 14.0)	–7.7 (−20.8, 7.7)	–11.7 (−24.0, 2.6)	****–**20.9 (−34.7, −4.2)**	–2.4 (−20.0, 19.0)	–1.8 (−17.2, 16.5)	16.6 (−18.4, 66.5)
OEC/UT	12.0 (−4.1, 30.7)	–20.0 (−38.3, 3.9)	–5.4 (−17.1, 8.0)	–13.6 (−25.7, 0.4)	****–**20.7 (−30.9, −8.9)**	****–**27.4 (−42.0, −9.1)**	5.1 (−18.3, 35.3)	–2.8 (−19.8, 17.8)	0.3 (−30.4, 44.6)
									
highest parental education									
less than college degree	ref	ref	ref	ref	ref	ref	ref	ref	ref
bachelor’s degree	0.7 (−10.1, 12.8)	0.7 (−17.5, 23.0)	8.8 (−2.0, 20.8)	–0.6 (−11.7, 11.8)	11.6 (−0.5, 25.2)	3.9 (−10.9, 21.1)	4.9 (−9.2, 21.1)	4.0 (−8.6, 18.3)	–4.5 (−28.3, 27.1)
graduate or professional	–10.6 (−22.4, 3.1)	5.2 (−13.6, 28.3)	12.4 (−0.4, 26.9)	–1.3 (−12.9, 11.8)	**21.1 (4.9, 39.8)**	18.6 (−1.7, 43.0)	16.4 (−5.7, 43.7)	**29.3 (7.9, 54.8)**	16.8 (−18.7, 68.0)
pooled *R*^2^ estimate^a^	0.10	0.16	0.33	0.23	0.18	0.14	0.20	0.28	0.20

a Computed by pooling *R*^2^ estimates of the regression models fitted to 20 multiply-imputed data sets.

b Note: Estimates with a *p*-value <0.05 are highlighted in bold. ASD, autism spectrum disorder; CHARGE, Childhood Autism Risks from Genetics and Environment; CI, confidence interval; DD, developmental delay; MeFOSAA, *N*-methyl perfluorooctane sulfonamido acetic acid; OEC/UT, other early concerns/unaffected twin; PFAS, per- and polyfluoroalkyl substances; PFDA, perfluorodecanoic acid; PFHpA, perfluoroheptanoic acid; PFHxS, perfluorohexane-1-sulfonic acid; PFNA, perfluorononanoic acid; PFOA, perfluorooctanoic acid; PFOS, perfluorooctanesulfonic acid; PFPeA, perfluoro-*n*-pentanoic acid; PFUnDA, perfluoroundecanoic acid; ref, reference group; *R*^2^, coefficient of determination; TD, typical development.

In the subset of 327 mother–child dyads, all PFAS concentrations decreased over time in a similar magnitude (−23 to −6%) (Table S4). When additionally adjusting for maternal serum concentrations in corresponding regression models for five PFAS, the results were slightly attenuated, with the magnitude of annual mean percent changes for PFOA, PFHxS, and PFNA decreased (Table S5). Increasing maternal serum concentrations of five PFAS were associated with higher concentrations of the corresponding PFAS in children (percent change per ln-transformed unit increase: 20% for PFOA, 31% for PFNA, 28% for PFDA, 60% for PFHxS, and 42% for PFOS). Breastfeeding duration remained as a major determinant for all five PFAS, with percent change per month ranging from 2 to 4%.

The *R*^2^ values of the regression models using 541 children were relatively higher for PFOA, PFNA, PFHxS, PFOS, and MeFOSAA (*R*^2^ = 0.20**–**0.33) than those for PFPeA, PFHpA, PFDA, and PFUnDA (*R*^2^ = 0.10**–**0.18) (Table 3). When the regression analyses were restricted to 327 mother–child dyads, the *R*^2^ values were similar, except that the *R*^2^ value for PFHxS increased from 0.20 to 0.28 (Table S4). When additionally including maternal serum concentrations in corresponding regression models for five PFAS, the greatest increase in the *R*^2^ values was observed for PFHxS (from 0.28 to 0.42), followed by PFDA (from 0.18 to 0.26), PFNA (from 0.21 to 0.29), PFOS (from 0.30 to 0.37), and PFOA (from 0.37 to 0.39) (Table S5).

## 4 Discussion

Among 541 Northern California children who participated in the CHARGE study at ages 2–5 years, serum concentrations of common long-alkyl-chain PFAS (PFOA, PFNA, PFHxS, and PFOS) and one short-alkyl-chain PFAS (PFHpA) decreased, on average, by 10 to 12% per year during 2009–2017. MeFOSAA, a precursor of PFOS, declined the most (25% per year), and two long-alkyl-chain PFAS (PFDA and PFUnDA) and one short-alkyl-chain PFAS (PFPeA) decreased less steeply (6–9% per year). Declines in child serum concentrations of common long-alkyl-chain PFAS and MeFOSAA over the study period likely reflect the phase-out of these compounds in consumer products. Less steep decreases in PFDA and PFUnDA concentrations can be attributed in part to less active regulation compared to common long-alkyl-chain PFAS or longer half-lives resulting from their slower elimination rates.^49−51^ The smallest decrease was observed for PFPeA, the shortest-alkyl-chain PFAS quantified, despite its shorter half-life.^52,53^ This finding might have been influenced by continued exposure arising from the substitution of long-alkyl-chain PFAS with short-alkyl-chain alternatives and ongoing use of fluorotelomer alcohols.^54,55^ However, as these declines were generally not linear, the average declines are, for most of the measured PFAS, representing a shorter-term steeper decline in the early years of this study, 2009–2011, followed by a flattening. From 2011 to 2016, concentrations of many PFAS either declined much more slowly or appeared to have leveled off.

There are limited studies investigating the temporal trends in PFAS concentrations in young children during comparable study periods.^31,35^ A study of the U.S. children aged 6–10 years observed that concentrations of PFOA, PFHxS, PFOS, and MeFOSAA decreased during 2007–2010, while those of PFNA increased.^35^ However, another study on 4 year-old Swedish children, who were consuming drinking water contaminated with PFBS, PFHxS, and PFOS, did not observe any noticeable temporal trends in concentrations of PFHpA, PFOA, PFNA, PFDA, PFHxS, and PFOS during 2008–2015.^31^ These inconsistent findings may be attributable to variations in sample sizes, differences in sources of exposure, sociodemographic characteristics, regulatory status, or differences in exposure patterns in different geographic locations. Serum concentrations of common long-alkyl-chain PFAS in our study children were comparable to those in most of the previous studies that used study populations with overlapping age ranges and time periods (Table S6).^14,22,24,25,31,32^ When compared to 3 to 5 year-old children from the nationally representative U.S. National Health and Nutrition Examination Survey (NHANES) 2013–2014,^24^ the CHARGE study children who provided serum samples at the same period had similar PFOA and PFNA and slightly lower PFHxS and PFOS concentrations.

Our previous study examined serum from 450 CHARGE mothers, including 327 mothers included in this study, to assess temporal trends of their serum PFAS concentrations during the same period.^56^ When adjusting for maternal race/ethnicity, age, education, homeownership, parity, prepregnancy body mass index, and breastfeeding duration, annual mean percent changes of serum PFOA and PFOS concentrations in maternal samples (−10.7 and −10.8% per year, respectively) were similar to those in the children’s samples described here. Maternal PFHxS concentrations also declined but less steeply (−8.0% per year) compared to child concentrations. On the other hand, there were mixed trends observed in maternal PFNA and PFDA concentrations, whereas child concentrations consistently decreased. These findings can be partially explained by different exposure sources or pathways for PFNA and PFDA between mothers and children. Female adults are exposed to PFAS primarily through dietary intake, while toddlers and preschoolers can be exposed to PFAS through hand-to-mouth transfers from nondietary sources, such as treated carpets, in addition to diet.^13^ According to the U.S. EPA, there was a reduction of long-alkyl-chain PFCAs, including PFNA, PFDA, and PFUnDA, in many commercial articles, including carpet, carpet/fabric-care liquids, household floor wax, and treated apparels and home textiles, during 2007–2011.^57^ Therefore, it is plausible that PFNA and PFDA levels present in treated carpets and furniture have decreased over time, leading to reduced levels of exposure of young children to these compounds.

In our 327 mother–child dyads, serum concentrations of PFOA, PFNA, and PFHxS were higher in child samples than in maternal samples, whereas those of PFOS were higher in maternal samples. Prior studies that quantified PFAS concentrations in young children and their matched mothers consistently reported higher concentrations of PFOA, PFNA, PFDA, and PFOS in children compared to their mothers.^28,38^ Increased PFAS concentrations in children can be attributed to exposures arising from higher water/food ingestion rates per body weight as well as nondietary exposures due to more frequent hand-to-mouth activities and closer proximity to the ground.^13,14,18^ Furthermore, it is plausible that maternal serum PFAS concentrations decreased from pregnancy to a few years postpartum as a result of gestational and/or lactational transfers and menstrual excretion,^58,59^ whereas young child PFAS concentrations increased due to breastfeeding.^16^

As expected, we observed in our study population that breastfeeding duration is a major determinant for maternal and child serum PFAS concentrations, showing negative correlations with maternal PFAS concentrations (*r*_sp_ = −0.37 to −0.16) and positive correlations with child PFAS concentrations (*r*_sp_ = 0.17–0.43) (Table S2). Increased child PFAS concentrations and decreased maternal PFAS concentrations, both influenced by breastfeeding duration, were likely to contribute to relatively weaker correlations between maternal and child PFAS concentrations (*r*_sp_ = 0.13–0.39). In comparison, a previous study in Norway showed strong correlations between prenatal maternal and 3 year-old child PFAS concentrations (*r*_sp_ = 0.50–0.66), despite the children being breastfed for an average of 12 months.^32^ In line with our findings, two previous studies observed relatively weak to moderate correlations of children aged 2–8 years or 6–11 years and their mothers (*r*_sp_ = 0.27–0.43 and 0.25–0.48, respectively).^28,38^ Another study showed stronger correlations between mothers and their 2 to 4 month-old infants (*r*_sp_ = 0.50–0.66),^37^ possibly influenced primarily by gestational transfer and less depletion of maternal PFAS concentrations resulting from shorter breastfeeding duration. MeFOSAA was the only exception, as neither maternal nor child concentrations in this study were correlated with breastfeeding duration. This resulted in strong correlations between maternal and child concentrations (*r*_sp_ = 0.70), consistent with previous findings.^28^ Nevertheless, it should be noted that maternal PFAS concentrations were significant determinants for child PFAS concentrations, which increased model *R*^2^ values. Potential contributing factors include long-term effects from gestational transfer^32^ and shared exposure sources with mothers within the home environment, such as food, drinking water, carpets, and furniture.^28^

Breastfeeding duration was not associated with PFPeA and MeFOSAA concentrations in children aged 2–5 years but was positively associated with other PFAS concentrations. Consistent with our results and with the exception of MeFOSAA, a recent U.S. study reported that all measured long-alkyl-chain PFAS in reproductive-aged Black women were negatively associated with breastfeeding duration.^60^ However, MeFOSAA concentrations in breast milk as well as its lactational transfer efficiency have been rarely studied due to the low detection frequency.^60−63^ A Korean study observed higher concentrations of PFPeA and lower concentrations of long-alkyl-chain PFAS in breast milk.^52^ Due to the shorter half-life of short-alkyl chain PFAS compared with long-alkyl-chain PFAS,^52,53^ it is likely that serum PFPeA concentrations in our children reflect more recent exposures. Consequently, this could have resulted in null associations between PFPeA concentrations and breastfeeding duration in spite of the higher transfer efficiency of short-alkyl-chain PFAS from maternal blood to breast milk.^63^

Sociodemographic factors that influenced serum PFAS concentrations in our study children included race/ethnicity and parental education. In line with our findings, previous studies observed higher PFAS concentrations in White children compared to Black or other racial/ethnic groups,^29,33,35^ and children from more educated families have higher PFAS concentrations.^35,64^ These factors may be related to differences in diet, lifestyle, and household characteristics. For example, repeated use of waterproof clothing, frequent consumption of fish and fast food, and the presence of rugs or carpets in a home, among other activities, can contribute to exposure in children.^28,29,35^ Additionally, as in prior studies, we observed negative associations between several PFAS and child body weight at sampling, suggesting a dilution effect.^35,65^

To our knowledge, this is one of the large-scale studies, notably the largest in the U.S., that quantified the PFAS concentration in the serum of toddlers and preschoolers. A strength of this study is that it is one of the few that examined temporal trends of PFAS concentrations in young children over almost a decade and reported determinants of 9 ubiquitously detected PFAS, including two short-alkyl-chain PFAS. We also quantified PFAS in maternal serum samples collected during the same study visit when children were 2–5 years of age, allowing virtually simultaneous blood draws for examining correlations between child and maternal PFAS concentrations. However, it is important to acknowledge several limitations. First, our regression models for five common long-alkyl-chain PFAS explained 26–42% of variability even after adjusting for maternal serum PFAS concentrations. We were unable to account for other major determinants of child PFAS concentrations, including dietary choices or habits, PFAS concentrations in drinking water and household dust, and prenatal PFAS concentrations.^28,31,32,35^ Second, over half of the children in our study had been diagnosed with ASD or DD, and childhood PFAS concentrations were associated with increased odds of ASD.^41^ Although we did adjust for the diagnostic group in the regression models and observed similar findings with other population-based studies, the generalizability of our findings might be limited. Third, this study used 327 mother–child dyads, comprising ASD and TD children only, to examine correlations and associations between maternal and child serum PFAS concentrations, which excluded children who were either developmentally delayed or had other disabilities that prevented their participation in CHARGE or the giving of blood samples and therefore may have introduced selection bias. Still, we confirmed no substantial differences in demographic characteristics and child PFAS concentrations between 541 children and the subset of 327 children. Last, PFAS concentrations in child and maternal serum samples were measured at different laboratories; differences in LODs could have potentially influenced the distribution of PFAS concentrations and their correlations.

In conclusion, our study observed a decrease in serum concentrations of select PFAS in 2 to 5 year-old children primarily from 2009 to 2011, with some PFAS concentrations continuing to decline through 2017. This decline can be attributed to voluntary phase-out efforts and regulatory measures targeting common long-alkyl-chain PFAS initiated in the early 2000s. The widespread detection (>95%) of two short-chain PFAS, PFPeA and PFHpA, in our children’s serum aligns with growing use of these compounds as substitutes for long-alkyl-chain PFAS.^55^ Further research can help explore their temporal trends after 2017 and exposure sources and pathways , especially in susceptible populations. Our findings also indicate that breastfeeding duration is a major determinant of most PFAS concentrations in child and maternal serum samples collected during the same study visit. Despite the transfer of PFAS from mother to child, particularly through breastfeeding, the correlations between child and maternal PFAS concentrations at ages 2–5 years were positive, although modest in magnitude. The positive results are at least partially explained by the shared exposure sources of children with their mothers even after breastfeeding ended.

## Supporting Information Available

Distribution of serum PFAS concentrations in 327 mother–child dyads (Table S1), Spearman correlation coefficients (*r*_sp_) between maternal and child serum PFAS concentrations and between maternal or child PFAS concentrations and breastfeeding duration in 327 mother–child dyads (Table S2), bivariate associations between serum PFAS concentrations and potential determinants in 541 children (Table S3), adjusted mean percent changes (95% CIs) in serum PFAS concentrations per one-unit increase of each potential determinant in 327 children whose mothers provided serum samples for PFAS quantification (Table S4), adjusted mean percent changes (95% CIs) in serum PFAS concentrations per one-unit increase of each potential determinant in 327 children whose mothers provided serum samples for PFAS quantification, additionally adjusting for maternal serum PFAS concentrations (Table S5), median PFAS concentrations (ng/mL) in children’s blood reported in previous studies that used study populations with overlapping age ranges and study periods (Table S6), Spearman correlation coefficients among 9 serum PFAS concentrations in 541 children (Figure S1), and comparison between maternal and child serum PFAS concentrations in 327 mother–child dyads (Figure S2) (PDF)

## Notes

The content is solely the responsibility of the authors and does not necessarily represent the official views of the National Institutes of Health. The findings and conclusions in this report are those of the authors and do not necessarily represent the official position of the Centers for Disease Control and Prevention (CDC). Use of trade name is for identification only and does not imply endorsement by the CDC, the Public Health Service, or the U.S. Department of Health and Human Services.

## Notes

The CHARGE study protocol and this study were approved by the institutional review boards for the State of California, the University of California Davis, and the University of Texas at Arlington. Participants provided written informed consent before collection of any data. The analysis of coded specimens at the Centers for Disease Control and Prevention (CDC) laboratory was determined by CDC not to constitute engagement in human subject research.

## References

[ref1] Lindstrom A. B.; Strynar M. J.; Libelo E. L. Polyfluorinated compounds: past, present, and future. Environmental science & technology. 2011, 45 (19), 7954–7961. 10.1021/es2011622. 21866930

[ref2] ATSDR. Toxicological Profile for Perfluoroalkyls. 2021. 37220203

[ref3] Prevedouros K.; Cousins I. T.; Buck R. C.; Korzeniowski S. H. Sources, fate and transport of perfluorocarboxylates. Environmental science & technology. 2006, 40 (1), 32–44. 10.1021/es0512475. 16433330

[ref4] Centers for Disease Control and Prevention, U.S. Department of Health and Human Services. National Report on Human Exposure to Environmental Chemicals. 2023. https://www.cdc.gov/exposurereport/.

[ref5] U.S. Environmental Protection Agency. Health Effects Support Document for Perfluorooctane Sulfonate (PFOS). 2016. EPA 822- R-16–002. https://www.epa.gov/sdwa/drinking-water-health-advisories-pfoa-and-pfos.

[ref6] U.S. Environmental Protection Agency. Health Effects Support Document for Perfluorooctanoic Acid (PFOA). 2016. EPA 822- R-16–003. https://www.epa.gov/sdwa/drinking-water-health-advisories-pfoa-and-pfos.

[ref7] Sunderland E. M.; Hu X. C.; Dassuncao C.; Tokranov A. K.; Wagner C. C.; Allen J. G. A review of the pathways of human exposure to poly-and perfluoroalkyl substances (PFASs) and present understanding of health effects. Journal of exposure science & environmental epidemiology. 2019, 29 (2), 131–147. 10.1038/s41370-018-0094-1. 30470793 PMC6380916

[ref8] U.S. Environmental Protection Agency. Technical Fact Sheet - Perfluorooctane Sulfonate (PFOS) and Perfluorooctanoic Acid (PFOA). 2017. EPA 505-F-17–001.

[ref9] Land M.; De Wit C. A.; Bignert A.; et al. What is the effect of phasing out long-chain per-and polyfluoroalkyl substances on the concentrations of perfluoroalkyl acids and their precursors in the environment? A systematic review. Environ. Evidence 2018, 7, 1–32. 10.1186/s13750-017-0114-y.

[ref10] U.S. Environmental Protection Agency. Fact Sheet: 2010/2015 PFOA Stewardship Program. 2023. https://www.epa.gov/assessing-and-managing-chemicals-under-tsca/fact-sheet-20102015-pfoa-stewardship-program.

[ref11] United Nations Environmental Program. Stockholm Convention on Persistent Organic Pollutants (POPs)—Text and annexes. Revised in 2019. 2023. http://chm.pops.int/Convention/ConventionText/tabid/2232.

[ref12] United Nations Environmental Program. All POPs listed in the Stockholm Convention. 2023. http://chm.pops.int/TheConvention/ThePOPs/AllPOPs/tabid/2509.

[ref13] Trudel D.; Horowitz L.; Wormuth M.; Scheringer M.; Cousins I. T.; Hungerbühler K. Estimating consumer exposure to PFOS and PFOA. Risk Analysis: An International Journal. 2008, 28 (2), 251–269. 10.1111/j.1539-6924.2008.01017.x. 18419647

[ref14] Zhang T.; Sun H. W.; Wu Q.; Zhang X. Z.; Yun S. H.; Kannan K. Perfluorochemicals in meat, eggs and indoor dust in China: assessment of sources and pathways of human exposure to perfluorochemicals. Environmental science & technology. 2010, 44 (9), 3572–3579. 10.1021/es1000159. 20377175

[ref15] Winkens K.; Vestergren R.; Berger U.; Cousins I. T. Early life exposure to per-and polyfluoroalkyl substances (PFASs): A critical review. Emerging Contaminants. 2017, 3 (2), 55–68. 10.1016/j.emcon.2017.05.001.

[ref16] Mogensen U. B.; Grandjean P.; Nielsen F.; Weihe P.; Budtz-Jørgensen E. Breastfeeding as an exposure pathway for perfluorinated alkylates. Environ. Sci. Technol. 2015, 49 (17), 10466–10473. 10.1021/acs.est.5b02237. 26291735 PMC6190571

[ref17] Mondal D.; Weldon R. H.; Armstrong B. G.; et al. Breastfeeding: a potential excretion route for mothers and implications for infant exposure to perfluoroalkyl acids. Environmental health perspectives. 2014, 122 (2), 187–192. 10.1289/ehp.1306613. 24280536 PMC3915259

[ref18] Landrigan P. J.; Miodovnik A. Children’s health and the environment: an overview. Mount Sinai Journal of Medicine: A Journal of Translational and Personalized Medicine. 2011, 78 (1), 1–10. 10.1002/msj.20236. 21259259

[ref19] Olsen G. W.; Church T. R.; Hansen K. J.; et al. Quantitative evaluation of perfluorooctanesulfonate (PFOS) and other fluorochemicals in the serum of children. J. Children’s Health 2004, 2 (1), 53–76. 10.1080/15417060490447378.

[ref20] Toms L-ML; Calafat A. M.; Kato K.; et al. Polyfluoroalkyl chemicals in pooled blood serum from infants, children, and adults in Australia. Environmental science & technology. 2009, 43 (11), 4194–4199. 10.1021/es900272u. 19569351

[ref21] Zhang T.; Wu Q.; Sun H. W.; Zhang X. Z.; Yun S. H.; Kannan K. Perfluorinated compounds in whole blood samples from infants, children, and adults in China. Environmental science & technology. 2010, 44 (11), 4341–4347. 10.1021/es1002132. 20441147

[ref22] Schecter A.; Malik-Bass N.; Calafat A. M.; et al. Polyfluoroalkyl compounds in Texas children from birth through 12 years of age. Environmental health perspectives. 2012, 120 (4), 590–594. 10.1289/ehp.1104325. 22182702 PMC3339466

[ref23] Zhang R.; Ye J.; Wei Q.; et al. Plasma concentration of 14 perfluoroalkyl acids (PFAAs) among children from seven cities in Guangdong, China. Sci. Total Environ. 2018, 616, 1469–1476. 10.1016/j.scitotenv.2017.10.167. 29066194

[ref24] Ye X.; Kato K.; Wong L.-Y.; et al. Per-and polyfluoroalkyl substances in sera from children 3 to 11 years of age participating in the National Health and Nutrition Examination Survey 2013–2014. International journal of hygiene and environmental health. 2018, 221 (1), 9–16. 10.1016/j.ijheh.2017.09.011. 28993126 PMC5726901

[ref25] Duffek A.; Conrad A.; Kolossa-Gehring M.; et al. Per-and polyfluoroalkyl substances in blood plasma–Results of the German Environmental Survey for children and adolescents 2014–2017 (GerES V). International Journal of Hygiene and Environmental Health. 2020, 228, 113549 10.1016/j.ijheh.2020.113549. 32502942

[ref26] Fromme H.; Mosch C.; Morovitz M.; et al. Pre-and postnatal exposure to perfluorinated compounds (PFCs). Environmental science & technology. 2010, 44 (18), 7123–7129. 10.1021/es101184f. 20722423

[ref27] Blomberg A. J.; Shih Y.-H.; Messerlian C.; Jo̷rgensen L. H.; Weihe P.; Grandjean P. Early-life associations between per-and polyfluoroalkyl substances and serum lipids in a longitudinal birth cohort. Environ. Res. 2021, 200, 111400 10.1016/j.envres.2021.111400. 34081971 PMC8403652

[ref28] Wu X. M.; Bennett D. H.; Calafat A. M.; et al. Serum concentrations of perfluorinated compounds (PFC) among selected populations of children and adults in California. Environmental research. 2015, 136, 264–273. 10.1016/j.envres.2014.09.026. 25460645 PMC4724210

[ref29] Kingsley S. L.; Eliot M. N.; Kelsey K. T.; et al. Variability and predictors of serum perfluoroalkyl substance concentrations during pregnancy and early childhood. Environmental research. 2018, 165, 247–257. 10.1016/j.envres.2018.04.033. 29734025 PMC6309672

[ref30] Koponen J.; Winkens K.; Airaksinen R.; et al. Longitudinal trends of per-and polyfluoroalkyl substances in children’s serum. Environment international. 2018, 121, 591–599. 10.1016/j.envint.2018.09.006. 30308470

[ref31] Gyllenhammar I.; Benskin J. P.; Sandblom O.; et al. Perfluoroalkyl acids (PFAAs) in children’s serum and contribution from PFAA-contaminated drinking water. Environmental science & technology. 2019, 53 (19), 11447–11457. 10.1021/acs.est.9b01746. 31476116

[ref32] Papadopoulou E.; Sabaredzovic A.; Namork E.; Nygaard U. C.; Granum B.; Haug L. S. Exposure of Norwegian toddlers to perfluoroalkyl substances (PFAS): the association with breastfeeding and maternal PFAS concentrations. Environment international. 2016, 94, 687–694. 10.1016/j.envint.2016.07.006. 27453094

[ref33] van Beijsterveldt I. A.; van Zelst B. D.; van den Berg S. A.; de Fluiter K. S.; van der Steen M.; Hokken-Koelega A. C. Longitudinal poly-and perfluoroalkyl substances (PFAS) levels in Dutch infants. Environment international. 2022, 160, 107068 10.1016/j.envint.2021.107068. 34968992

[ref34] Spliethoff H. M.; Tao L.; Shaver S. M.; et al. Use of newborn screening program blood spots for exposure assessment: declining levels of perfluorinated compounds in New York State infants. Environmental science & technology. 2008, 42 (14), 5361–5367. 10.1021/es8006244. 18754394

[ref35] Harris M. H.; Rifas-Shiman S. L.; Calafat A. M.; et al. Predictors of per-and polyfluoroalkyl substance (PFAS) plasma concentrations in 6–10 year old American children. Environmental science & technology. 2017, 51 (9), 5193–5204. 10.1021/acs.est.6b05811. 28325044 PMC5576362

[ref36] Thomsen C.; Haug L. S.; Stigum H.; Fro̷shaug M.; Broadwell S. L.; Becher G. Changes in concentrations of perfluorinated compounds, polybrominated diphenyl ethers, and polychlorinated biphenyls in Norwegian breast-milk during twelve months of lactation. Environ. Sci. Technol. 2010, 44 (24), 9550–9556. 10.1021/es1021922. 21090747

[ref37] Gyllenhammar I.; Benskin J. P.; Sandblom O.; et al. Perfluoroalkyl acids (PFAAs) in serum from 2–4-month-old infants: Influence of maternal serum concentration, gestational age, breast-feeding, and contaminated drinking water. Environmental science & technology. 2018, 52 (12), 7101–7110. 10.1021/acs.est.8b00770. 29758986

[ref38] Mo̷rck T. A.; Nielsen F.; Nielsen J. K.; Siersma V. D.; Grandjean P.; Knudsen L. E. PFAS concentrations in plasma samples from Danish school children and their mothers. Chemosphere 2015, 129, 203–209. 10.1016/j.chemosphere.2014.07.018. 25147004

[ref39] Hertz-Picciotto I.; Croen L. A.; Hansen R.; Jones C. R.; Van de Water J.; Pessah I. N. The CHARGE study: an epidemiologic investigation of genetic and environmental factors contributing to autism. Environmental health perspectives. 2006, 114 (7), 1119–1125. 10.1289/ehp.8483. 16835068 PMC1513329

[ref40] Honda M.; Robinson M.; Kannan K. A rapid method for the analysis of perfluorinated alkyl substances in serum by hybrid solid-phase extraction. Environmental Chemistry. 2018, 15 (2), 92–99. 10.1071/EN17192.

[ref41] Oh J.; Shin H.-M.; Kannan K.; et al. Childhood exposure to per-and polyfluoroalkyl substances and neurodevelopment in the CHARGE case-control study. Environmental Research. 2022, 215, 114322 10.1016/j.envres.2022.114322. 36108719 PMC9976729

[ref42] Kato K.; Basden B. J.; Needham L. L.; Calafat A. M. Improved selectivity for the analysis of maternal serum and cord serum for polyfluoroalkyl chemicals. Journal of chromatography A 2011, 1218 (15), 2133–2137. 10.1016/j.chroma.2010.10.051. 21084089

[ref43] Hornung R. W.; Reed L. D. Estimation of average concentration in the presence of nondetectable values. Applied occupational and environmental hygiene. 1990, 5 (1), 46–51. 10.1080/1047322X.1990.10389587.

[ref44] Azur M. J.; Stuart E. A.; Frangakis C.; Leaf P. J. Multiple imputation by chained equations: what is it and how does it work?. International journal of methods in psychiatric research. 2011, 20 (1), 40–49. 10.1002/mpr.329. 21499542 PMC3074241

[ref45] White I. R.; Royston P.; Wood A. M. Multiple imputation using chained equations: issues and guidance for practice. Statistics in medicine. 2011, 30 (4), 377–399. 10.1002/sim.4067. 21225900

[ref46] Daoud J. I.. Multicollinearity and regression analysis. IOP Publishing; 2017: 012009 10.1088/1742-6596/949/1/012009.

[ref47] Zota A. R.; Calafat A. M.; Woodruff T. J. Temporal trends in phthalate exposures: findings from the National Health and Nutrition Examination Survey, 2001–2010. Environmental health perspectives. 2014, 122 (3), 235–241. 10.1289/ehp.1306681. 24425099 PMC3948032

[ref48] Lenth R.; Lenth M. R. Package ‘lsmeans’. Am. Stat. 2018, 34 (4), 216–221.

[ref49] Zhang Y.; Beesoon S.; Zhu L.; Martin J. W. Biomonitoring of perfluoroalkyl acids in human urine and estimates of biological half-life. Environmental science & technology. 2013, 47 (18), 10619–10627. 10.1021/es401905e. 23980546

[ref50] Drew R.; Hagen T. G.; Champness D.; Sellier A. Half-lives of several polyfluoroalkyl substances (PFAS) in cattle serum and tissues. Food Additives & Contaminants: Part A 2022, 39 (2), 320–340. 10.1080/19440049.2021.1991004. 34732107

[ref51] Ohmori K.; Kudo N.; Katayama K.; Kawashima Y. Comparison of the toxicokinetics between perfluorocarboxylic acids with different carbon chain length. Toxicology. 2003, 184 (2–3), 135–140. 10.1016/S0300-483X(02)00573-5. 12499116

[ref52] Kang H.; Choi K.; Lee H.-S.; et al. Elevated levels of short carbon-chain PFCAs in breast milk among Korean women: Current status and potential challenges. Environmental research. 2016, 148, 351–359. 10.1016/j.envres.2016.04.017. 27111244

[ref53] Xu Y.; Fletcher T.; Pineda D.; et al. Serum half-lives for short-and long-chain perfluoroalkyl acids after ceasing exposure from drinking water contaminated by firefighting foam. Environ. Health Perspect. 2020, 128 (7), 077004 10.1289/EHP6785. 32648786 PMC7351026

[ref54] Chang C.-J.; Ryan P. B.; Smarr M. M.; et al. Serum per-and polyfluoroalkyl substance (PFAS) concentrations and predictors of exposure among pregnant African American women in the Atlanta area. Georgia. Environmental research. 2021, 198, 110445 10.1016/j.envres.2020.110445. 33186575 PMC8107192

[ref55] Ateia M.; Maroli A.; Tharayil N.; Karanfil T. The overlooked short-and ultrashort-chain poly-and perfluorinated substances: A review. Chemosphere. 2019, 220, 866–882. 10.1016/j.chemosphere.2018.12.186. 33395808

[ref56] Kim K.; Bennett D. H.; Calafat A. M.; Hertz-Picciotto I.; Shin H.-M. Temporal trends and determinants of serum concentrations of per-and polyfluoroalkyl substances among Northern California mothers with a young child, 2009–2016. Environmental research. 2020, 186, 109491 10.1016/j.envres.2020.109491. 32361076 PMC7363519

[ref57] Liu X., Guo Z., Krebs K. A., Pope R. H., Roache N. F. Trends of Perfluoroalkyl Acid Content in Articles of Commerce—Market Monitoring from 2007 through 2011. 2012 EPA. EPA/600/R-12/585.

[ref58] Oh J.; Bennett D. H.; Tancredi D. J.; et al. Longitudinal Changes in Maternal Serum Concentrations of Per-and Polyfluoroalkyl Substances from Pregnancy to Two Years Postpartum. Environmental Science & Technology. 2022, 56 (16), 11449–11459. 10.1021/acs.est.1c07970. 35904360 PMC9798824

[ref59] Upson K.; Shearston J. A.; Kioumourtzoglou M.-A. An epidemiologic review of menstrual blood loss as an excretion route for per-and polyfluoroalkyl substances. Current environmental health reports. 2022, 9 (1), 29–37. 10.1007/s40572-022-00332-0. 35267175 PMC9876536

[ref60] Wise L. A.; Wesselink A. K.; Schildroth S.; et al. Correlates of plasma concentrations of per-and poly-fluoroalkyl substances among reproductive-aged Black women. Environmental research. 2022, 203, 111860 10.1016/j.envres.2021.111860. 34403666 PMC8616815

[ref61] Awad R.; Zhou Y.; Nyberg E.; et al. Emerging per-and polyfluoroalkyl substances (PFAS) in human milk from Sweden and China. Environmental Science: Processes & Impacts. 2020, 22 (10), 2023–2030. 10.1039/D0EM00077A. 32940316

[ref62] Nyberg E.; Awad R.; Bignert A.; Ek C.; Sallsten G.; Benskin J. P. Inter-individual, inter-city, and temporal trends of per-and polyfluoroalkyl substances in human milk from Swedish mothers between 1972 and 2016. Environmental Science: Processes & Impacts. 2018, 20 (8), 1136–1147. 10.1039/C8EM00174J. 29987291

[ref63] Zheng P.; Liu Y.; An Q.; et al. Prenatal and postnatal exposure to emerging and legacy per-/polyfluoroalkyl substances: Levels and transfer in maternal serum, cord serum, and breast milk. Science of The Total Environment. 2022, 812, 152446 10.1016/j.scitotenv.2021.152446. 34952085

[ref64] Montazeri P.; Thomsen C.; Casas M.; et al. Socioeconomic position and exposure to multiple environmental chemical contaminants in six European mother-child cohorts. International journal of hygiene and environmental health. 2019, 222 (5), 864–872. 10.1016/j.ijheh.2019.04.002. 31010791 PMC8713641

[ref65] Kim D.-H.; Lee M.-Y.; Oh J.-E. Perfluorinated compounds in serum and urine samples from children aged 5–13 years in South Korea. Environ. Pollut. 2014, 192, 171–178. 10.1016/j.envpol.2014.05.024. 24952613

