# Supplementary material for: Per- and Polyfluoroalkyl Substances (PFAS) in Serum of 2 to 5 year-Old Children: Temporal Trends, Determinants, and Correlations with Maternal PFAS Concentrations
Source: Environ Sci Technol. 2024 Feb 9;58(7):3151–62. doi: 10.1021/acs.est.3c08928 (PMC10882966; doi:10.1021/acs.est.3c08928)
Supplement: Supplementary file 1 — es3c08928_si_001.pdf [file es3c08928_si_001.pdf]

Supporting Information

Per- and polyfluoroalkyl substances (PFAS) in serum of 2- to 5-year-old children: temporal trends, determinants, and correlations with maternal PFAS concentrations

Jiwon Oh, Hyeong-Moo Shin, Kurunthachalam Kannan, Antonia M. Calafat, Rebecca J. Schmidt, Irva Hertz-Picciotto, Deborah H. Bennett

Pages: 9

Figures: 2

Tables: 6

Table S1. Distribution of serum PFAS concentrations in 327 CHARGE mother-child dyads.

Table S2. Spearman correlation coefficients (r_{sp}) between maternal and child serum PFAS concentrations and between maternal or child PFAS concentrations and breastfeeding duration in 327 CHARGE mother-child dyads.

Table S3. Bivariate associations between serum PFAS concentrations and potential determinants in 541 CHARGE children.

Table S4. Adjusted mean percent changes (95% CIs) in serum PFAS concentrations per one-unit increase of each potential determinant in 327 CHARGE children whose mothers provided serum samples for PFAS quantification.

Table S5. Adjusted mean percent changes (95% CIs) in serum PFAS concentrations per one-unit increase of each potential determinant in 327 CHARGE children whose mothers provided serum samples for PFAS quantification, additionally adjusting for maternal serum PFAS concentrations.

Table S6. Median PFAS concentrations (ng/mL) in children's blood reported in previous studies that used study populations with overlapping age ranges and study periods.

Figure S1. Spearman correlation coefficients among 9 serum PFAS concentrations in 541 CHARGE children.

Figure S2. Distributions of maternal and child serum PFAS concentrations in 327 CHARGE mother-child dyads.

Table S1. Distribution of serum PFAS concentrations in 327 CHARGE mother-child dyads.

PFAS	LOD (ng/mL)	% detection	Percentiles (ng/mL) 5th	25th	50th	75th	95th
Child serum							
PFPeA	0.05	99.4	0.22	0.37	0.55	0.76	1.20
PFHxA	0.05	14.1	<LOD	<LOD	<LOD	<LOD	0.24
PFHpA	0.02	96.3	0.04	0.13	0.22	0.38	0.85
PFOA	0.05	100.0	0.96	1.74	2.54	3.78	6.57
PFNA	0.02	100.0	0.34	0.58	0.85	1.27	3.03
PFDA	0.02	99.7	0.07	0.11	0.16	0.24	0.50
PFUnDA	0.02	77.4	<LOD	0.02	0.04	0.07	0.16
PFDoDA	0.05	5.8	<LOD	<LOD	<LOD	<LOD	0.06
PFBS	0.02	28.4	<LOD	<LOD	<LOD	0.02	0.08
PFHxS	0.02	100.0	0.25	0.39	0.66	1.10	3.11
PFOS	0.02	100.0	0.98	1.70	2.45	3.90	10.70
FOSA	0.02	0.6	<LOD	<LOD	<LOD	<LOD	<LOD
MeFOSAA	0.02	81.3	<LOD	0.04	0.11	0.32	1.59
EtFOSAA	0.02	16.2	<LOD	<LOD	<LOD	<LOD	0.07
Maternal serum							
PFOA	0.1	100.0	0.47	0.67	1.12	1.67	3.41
PFNA	0.1	94.2	<LOD	0.40	0.50	0.70	1.27
PFDA	0.1	68.2	<LOD	<LOD	0.20	0.30	0.50
PFUnDA	0.1	35.8	<LOD	<LOD	<LOD	0.10	0.30
PFDoDA	0.1	2.8	<LOD	<LOD	<LOD	<LOD	<LOD
PFHxS	0.1	97.6	0.20	0.30	0.50	0.70	1.50
PFOS	0.1	100.0	1.20	2.20	3.30	5.10	9.95
MeFOSAA	0.1	46.2	<LOD	<LOD	<LOD	0.20	0.50
EtFOSAA	0.1	0.3	<LOD	<LOD	<LOD	<LOD	<LOD

Note: CHARGE, Childhood Autism Risks from Genetics and Environment; FOSA, perfluorooctanesulfonamide; EtFOSAA, N-ethyl perfluorooctane sulfonamido acetic acid; LOD, limit of detection; MeFOSAA, N-methyl perfluorooctane sulfonamido acetic acid; PFAS, per- and polyfluoroalkyl substances; PFBS, perfluorobutanesulfonic acid; PFCA, perfluoroalkyl carboxylic acids; PFDA, perfluorodecanoic acid; PFHpA, perfluoroheptanoic acid; PFDoDA, perfluorododecanoic acid; PFHxA, perfluorohexanoic acid; PFHxS, perfluorohexane-1-sulfonic acid; PFNA, perfluorononanoic acid; PFOA, perfluorooctanoic acid; PFOS, perfluorooctanesulfonic acid; PFPeA, perfluoro-n-pentanoic acid; PFSA, perfluoroalkane sulfonic acids; PFUnDA, perfluoroundecanoic acid.

Table S2. Spearman correlation coefficients (r_{sp}) between maternal and child serum PFAS concentrations and between maternal or child PFAS concentrations and breastfeeding duration in 327 CHARGE mother-child dyads.

	**Maternal PFAS & child PFAS**		**Child PFAS & breastfeeding duration**		**Maternal PFAS & breastfeeding duration**	
**PFAS**	**N [a]**	r_{sp}	**N [b]**	r_{sp}	**N [b]**	r_{sp}
PFOA	327	0.13	507	0.43	310	-0.37
PFNA	308	0.31	507	0.22	294	-0.25
PFDA	222	0.39	505	0.23	213	-0.16
PFUnDA	98	0.38	376	0.17	112	0.05 [c]
PFHxS	319	0.36	507	0.35	303	-0.20
PFOS	327	0.26	507	0.34	310	-0.24
MeFOSAA	139	0.68	412	-0.06 [c]	142	0.05 [c]

Note: CHARGE, Childhood Autism Risks from Genetics and Environment; MeFOSAA, N-methyl perfluorooctane sulfonamido acetic acid; PFAS, per- and polyfluoroalkyl substances; PFDA, perfluorodecanoic acid; PFHxS, perfluorohexane-1-sulfonic acid; PFNA, perfluorononanoic acid; PFOA, perfluorooctanoic acid; PFOS, perfluorooctanesulfonic acid; PFUnDA, perfluoroundecanoic acidl r_{sp}, Spearman correlation coefficient.

[a] Correlation coefficients were calculated only when both maternal and child serum PFAS concentrations were detectable.

[b] Correlation coefficients were calculated only when maternal or child serum PFAS concentrations were detectable and breastfeeding duration was not missing.

[c] Statistically not significant (p>0.05).

Table S3. Bivariate associations between serum PFAS concentrations and potential determinants in 541 CHARGE children.

	Spearman correlation coefficients or median PFAS concentrations (25%, 75% percentiles)								
Potential determinants	PFPeA	PFHpA	PFOA	PFNA	PFDA	PFUnDA	PFHxS	PFOS	MeFOSAA
Sampling year (year)	**-0.29**	**-0.24**	**-0.40**	**-0.41**	**-0.31**	**-0.26**	**-0.38**	**-0.40**	**-0.43**
Child age at sampling (month)	**0.09**	**-0.23**	**-0.12**	-0.02	-0.07	0.00	**-0.09**	**-0.09**	**-0.10**
Child body weight at sampling (kg)	0.00	**-0.13**	**-0.12**	**-0.09**	**-0.09**	-0.05	**-0.10**	**-0.13**	**-0.12**
Maternal age at delivery (year)	-0.04	0.02	**0.09**	0.00	**0.10**	0.07	0.02	0.07	0.00
Breastfeeding duration (month)	-0.02	**0.14**	**0.43**	**0.22**	**0.23**	**0.17**	**0.35**	**0.34**	-0.06
Child race/ethnicity									
Non-Hispanic white	**0.5 (0.4, 0.8)**	0.2 (0.1, 0.4)	**2.7 (1.8, 4.0)**	**0.9 (0.6, 1.3)**	**0.2 (0.1, 0.3)**	0.0 (0.0, 0.1)	0.7 (0.4, 1.2)	**2.6 (1.8, 4.8)**	0.1 (0.0, 0.4)
Hispanic	**0.6 (0.4, 0.8)**	0.2 (0.1, 0.4)	**2.2 (1.6, 3.3)**	**0.9 (0.6, 1.3)**	**0.2 (0.1, 0.2)**	0.0 (0.0, 0.1)	0.6 (0.4, 1.0)	**2.5 (1.6, 3.8)**	0.1 (0.0, 0.3)
Black/Asian/multiracial	**0.5 (0.4, 0.7)**	0.2 (0.1, 0.3)	**2.1 (1.5, 3.5)**	**0.7 (0.4, 1.1)**	**0.1 (0.1, 0.2)**	0.0 (0.0, 0.1)	0.6 (0.3, 1.2)	**2.1 (1.3, 3.8)**	0.1 (0.0, 0.3)
Diagnostic group									
TD	**0.6 (0.4, 0.7)**	**0.2 (0.1, 0.4)**	2.6 (1.9, 4.0)	**0.9 (0.6, 1.4)**	**0.2 (0.1, 0.3)**	**0.1 (0.0, 0.1)**	**0.7 (0.5, 1.2)**	**2.6 (1.9, 4.1)**	**0.1 (0.1, 0.4)**
ASD	**0.5 (0.4, 0.8)**	**0.2 (0.1, 0.4)**	2.2 (1.6, 3.5)	**0.7 (0.5, 1.1)**	**0.1 (0.1, 0.2)**	**0.0 (0.0, 0.1)**	**0.6 (0.3, 1.0)**	**2.0 (1.3, 3.4)**	**0.1 (0.0, 0.3)**
DD	**0.6 (0.4, 0.9)**	**0.2 (0.1, 0.4)**	2.2 (1.5, 4.0)	**0.9 (0.6, 1.4)**	**0.1 (0.1, 0.3)**	**0.0 (0.0, 0.1)**	**0.6 (0.4, 1.1)**	**2.6 (1.6, 4.7)**	**0.1 (0.1, 0.5)**
OEC/UT	**0.7 (0.5, 0.8)**	**0.2 (0.1, 0.3)**	2.1 (1.6, 3.7)	**0.9 (0.7, 1.2)**	**0.1 (0.1, 0.2)**	**0.0 (0.0, 0.1)**	**0.8 (0.5, 1.3)**	**2.9 (1.7, 4.8)**	**0.1 (0.1, 0.3)**
Highest parental education									
Less than college degree	0.6 (0.4, 0.8)	0.2 (0.1, 0.4)	**2.1 (1.5, 3.5)**	0.9 (0.6, 1.3)	**0.1 (0.1, 0.2)**	0.0 (0.0, 0.1)	0.6 (0.4, 1.0)	**2.2 (1.4, 3.6)**	0.1 (0.0, 0.4)
Bachelor's degree	0.6 (0.4, 0.8)	0.2 (0.1, 0.4)	**2.6 (1.8, 3.8)**	0.8 (0.6, 1.2)	**0.2 (0.1, 0.3)**	0.0 (0.0, 0.1)	0.7 (0.4, 1.1)	**2.4 (1.7, 4.0)**	0.1 (0.0, 0.3)
Graduate or professional	0.5 (0.3, 0.8)	0.2 (0.1, 0.4)	**3.1 (2.0, 4.3)**	0.9 (0.6, 1.3)	**0.2 (0.1, 0.3)**	0.1 (0.0, 0.1)	0.8 (0.4, 1.3)	**3.4 (1.8, 5.8)**	0.1 (0.0, 0.5)
Homeownership									
Non-owner	0.6 (0.4, 0.8)	0.2 (0.1, 0.4)	**2.1 (1.5, 3.5)**	0.9 (0.6, 1.4)	**0.1 (0.1, 0.2)**	0.0 (0.0, 0.1)	0.6 (0.4, 1.1)	2.4 (1.5, 3.9)	0.1 (0.0, 0.4)
Owner	0.5 (0.4, 0.8)	0.2 (0.1, 0.4)	**2.6 (1.8, 3.7)**	0.8 (0.6, 1.2)	**0.2 (0.1, 0.3)**	0.0 (0.0, 0.1)	0.6 (0.4, 1.2)	2.5 (1.7, 4.2)	0.1 (0.0, 0.3)

Note: Estimates with a *p*-value<0.05 from the Spearman correlation test, Wilcoxon rank-sum test, or Kruskal-Wallis test are highlighted in bold. ASD, autism spectrum disorder; CHARGE, Childhood Autism Risks from Genetics and Environment; DD, developmental delay; MeFOSAA, N-methyl perfluorooctane sulfonamido acetic acid; OEC/UT, other early concerns/unaffected twin; PFAS, per- and polyfluoroalkyl substances; PFDA, perfluorodecanoic acid; PFHpA, perfluoroheptanoic acid; PFHxS, perfluorohexane-1-sulfonic acid; PFNA, perfluorononanoic acid; PFOA, perfluorooctanoic acid; PFOS, perfluorooctanesulfonic acid; PFPeA, perfluoro-n-pentanoic acid; PFUnDA, perfluoroundecanoic acid; TD, typical development.

Table S4. Adjusted mean percent changes (95% CIs) in serum PFAS concentrations per one-unit increase of each potential determinant in 327 CHARGE children whose mothers provided serum samples for PFAS quantification.

Potential determinants	PFPeA	PFHpA	PFOA	PFNA	PFDA	PFUnDA	PFHxS	PFOS	MeFOSAA
Sampling year (year)	**-5.5 (-8.2, -2.7)**	**-10.4 (-14.3, -6.3)**	**-9.3 (-11.5, -6.9)**	**-9.1 (-11.9, -6.3)**	**-5.6 (-8.2, -2.8)**	**-8.1 (-11.7, -4.4)**	**-11.7 (-14.7, -8.6)**	**-8.9 (-11.7, -5.9)**	**-23.0 (-28.3, -17.4)**
Child age at sampling (month)	**1.1 (0.3, 2.0)**	**-1.7 (-2.9, -0.4)**	-0.3 (-1.0, 0.4)	0.0 (-0.9, 0.9)	0.0 (-0.8, 0.9)	0.1 (-1.0, 1.3)	-0.3 (-1.3, 0.7)	-0.6 (-1.5, 0.3)	-1.3 (-3.3, 0.7)
Child body weight at sampling (kg)	0.3 (-2.1, 2.8)	-1.5 (-5.1, 2.2)	-1.1 (-3.2, 1.0)	-0.9 (-3.4, 1.7)	-1.3 (-3.6, 1.1)	-1.0 (-4.2, 2.3)	-1.6 (-4.4, 1.2)	**-2.8 (-5.3, -0.2)**	-4.4 (-9.8, 1.3)
Breastfeeding duration (month)	-0.6 (-1.4, 0.2)	**1.9 (0.7, 3.1)**	**3.2 (2.5, 3.9)**	**1.8 (1.0, 2.6)**	**1.9 (1.1, 2.6)**	1.0 (0.0, 2.1)	**3.2 (2.3, 4.2)**	**2.9 (2.1, 3.8)**	-0.4 (-2.3, 1.5)
Child race/ethnicity									
Non-Hispanic white	Ref	Ref	Ref	Ref	Ref	Ref	Ref	Ref	Ref
Hispanic	5.6 (-9.2, 22.8)	-2.1 (-22.4, 23.5)	-8.8 (-20.0, 4.0)	0.8 (-14.1, 18.3)	-4.9 (-18.1, 10.4)	0.1 (-18.4, 22.9)	-11.2 (-25.6, 6.1)	-6.2 (-20.4, 10.6)	**-30.9 (-52.1, -0.2)**
Black/Asian/multiracial	-5.1 (-19.0, 11.1)	**-21.7 (-38.6, -0.2)**	**-13.8 (-24.9, -1.1)**	**-16.3 (-29.2, -1.1)**	-14.3 (-26.7, 0.2)	-7.5 (-25.4, 14.6)	-3.8 (-20.1, 15.9)	-13.3 (-27.0, 3.0)	-15.2 (-42.2, 24.5)
Diagnostic group									
TD	Ref	Ref	Ref	Ref	Ref	Ref	Ref	Ref	Ref
ASD	0.8 (-11.5, 14.8)	**27.2 (4.1, 55.5)**	**14.4 (2.1, 28.2)**	-5.4 (-17.6, 8.5)	-7.4 (-18.6, 5.3)	**-24.7 (-36.9, -10.1)**	**16.7 (0.1, 36.0)**	1.5 (-11.9, 16.9)	12.3 (-17.9, 53.8)
Highest parental education									
Less than college degree	Ref	Ref	Ref	Ref	Ref	Ref	Ref	Ref	Ref
Bachelor's degree	-3.5 (-16.1, 11.0)	-10.6 (-27.9, 11.0)	6.8 (-5.5, 20.7)	-2.9 (-16.3, 12.6)	5.9 (-7.9, 21.6)	7.7 (-11.0, 30.4)	15.5 (-2.1, 36.3)	10.9 (-4.8, 29.2)	3.6 (-26.2, 45.3)
Graduate or professional	**-21.0 (-34.2, -5.3)**	2.8 (-22.4, 36.1)	10.4 (-5.8, 29.4)	-1.2 (-18.6, 19.8)	**21.9 (1.8, 46.0)**	**29.8 (1.3, 66.4)**	9.8 (-11.4, 36.1)	**26.2 (3.5, 53.8)**	21.8 (-21.6, 89.3)
Pooled R^2 estimate [a]	0.10	0.18	0.37	0.21	0.18	0.14	0.28	0.30	0.20

Note: Estimates with a p-value<0.05 are highlighted in bold. ASD, autism spectrum disorder; CHARGE, Childhood Autism Risks from Genetics and Environment; CI, confidence interval; MeFOSAA, N-methyl perfluorooctane sulfonamido acetic acid; PFAS, per- and polyfluoroalkyl substances; PFDA, perfluorodecanoic acid; PFHpA, perfluoroheptanoic acid; PFHxS, perfluorohexane-1-sulfonic acid; PFNA, perfluorononanoic acid; PFOA, perfluorooctanoic acid; PFOS, perfluorooctanesulfonic acid; PFPeA, perfluoro-n-pentanoic acid; PFUnDA, perfluoroundecanoic acid; Ref, reference group; R^2, coefficient of determination; TD, typical development.

[a] Computed by pooling R^2 estimates of the regression models fitted to 20 multiply imputed datasets.

Table S5. Adjusted mean percent changes (95% CIs) in serum PFAS concentrations per one-unit increase of each potential determinant in 327 CHARGE children whose mothers provided serum samples for PFAS quantification, additionally adjusting for maternal serum PFAS concentrations.

Potential determinants	PFOA	PFNA	PFDA	PFHxS	PFOS
Sampling year (year)	**-7.3 (-9.9, -4.7)**	**-8.9 (-11.6, -6.2)**	**-5.5 (-8.1, -2.9)**	**-8.3 (-11.2, -5.3)**	**-5.3 (-8.4, -2.2)**
Maternal serum PFAS [a]	**19.5 (7.5, 32.9)**	**31.3 (19.8, 43.8)**	**27.8 (17.6, 38.9)**	**59.9 (43.5, 78.3)**	**41.5 (26.0, 58.8)**
Child age at sampling (month)	-0.3 (-1.0, 0.5)	0.1 (-0.8, 0.9)	0.0 (-0.8, 0.8)	-0.1 (-1.0, 0.8)	-0.5 (-1.4, 0.4)
Child body weight at sampling (kg)	-1.1 (-3.1, 1.0)	-0.9 (-3.3, 1.6)	-0.8 (-3.0, 1.5)	-1.7 (-4.2, 0.9)	**-2.2 (-4.6, -0.3)**
Breastfeeding duration (month)	**3.7 (2.9, 4.4)**	**2.4 (1.5, 3.2)**	**2.2 (1.4, 2.9)**	**4.0 (3.2, 4.9)**	**3.8 (2.9, 4.6)**
Child race/ethnicity					
Non-Hispanic white	Ref	Ref	Ref	Ref	Ref
Hispanic	-5.4 (-17.0, 7.9)	4.5 (-10.3, 21.7)	-5.2 (-17.7, 9.3)	2.5 (-13.0, 20.7)	-0.3 (-14.9, 16.8)
Black/Asian/multiracial	-11.4 (-22.7, 1.6)	-14.2 (-26.9, 0.6)	**-15.1 (-26.8, -1.5)**	4.1 (-12.1, 23.3)	-9.4 (-23.1, 6.8)
Diagnostic group					
TD	Ref	Ref	Ref	Ref	Ref
ASD	**13.6 (1.5, 27.0)**	-4.3 (-16.0, 9.2)	-8.0 (-18.6, 4.0)	5.6 (-8.2, 21.5)	-1.7 (-14.1, 12.6)
Highest parental education					
Less than college degree	Ref	Ref	Ref	Ref	Ref
Bachelor's degree	6.1 (-5.9, 19.7)	-2.8 (-15.6, 12.0)	2.8 (-10.0, 17.3)	15.5 (-0.6, 34.1)	6.9 (-7.6, 23.7)
Graduate or professional	9.5 (-6.3, 28.0)	-5.3 (-21.2, 13.9)	9.6 (-8.1, 30.6)	4.6 (-13.8, 27.1)	14.8 (-5.1, 39.0)
Pooled R^2 estimate [b]	0.39	0.29	0.26	0.42	0.37

Note: Estimates with a *p*-value<0.05 are highlighted in bold. ASD, autism spectrum disorder; CHARGE, Childhood Autism Risks from Genetics and Environment; CI, confidence interval; PFAS, per- and polyfluoroalkyl substances; PFDA, perfluorodecanoic acid; PFHxS, perfluorohexane-1-sulfonic acid; PFNA, perfluorononanoic acid; PFOA, perfluorooctanoic acid; PFOS, perfluorooctanesulfonic acid; Ref, reference group; R^2, coefficient of determination; TD, typical development.

[a] Maternal serum PFAS concentrations below the limit of detection (0.1 ng/mL for all five PFAS) were multiply imputed. PFAS concentrations were natural log-transformed prior to regression analysis.

[b] Computed by pooling R^2 estimates of the regression models fitted to 20 multiply imputed datasets.

Table S6. Median PFAS concentrations (ng/mL) in children's blood reported in previous studies that used study populations with overlapping age ranges and study periods.

	**This study (*n*=541)**	**This study (*n*=100)**	**Schecter et al. 2012[1] (*n*=75)**	**Schecter et al. 2012[1] (*n*=75)**	**Zhang et al. 2010[2] (*n*=85)**	**Gyllenhammar et al. 2019[3] (*n*=57)**	**Papadopoulou et al. 2016[4] (*n*=112)**	**Ye et al. 2018[5] (*n*=181)**	**Duffek et al. 2020[6] (*n*=196)**	**Duffek et al. 2020[6] (*n*=491)**
Country	US (California)	US (California)	US (Texas)	US (Texas)	China	Sweden	Norway	US (NHANES)	Germany (GerES V)	Canada (CHMS)
Period	2009-2017	2013-2014	2009	2009	2009	2008-2015	2010-2011	2013-2014	2014-2017	2016-2027
Age	2-5	2-5	0-2	3-5	1-5	4	3	3-5	3-5	3-5
Matrix	Serum	Serum	Serum	Serum	Whole	Serum	Serum	Serum	Plasma	Plasma
PFPeA	0.57	0.41								
PFHxA	<0.05	<0.05								
PFHpA	0.21	0.18				0.12				
PFOA	2.38	1.88	2.00	3.10	2.42	2.50	2.63	1.80	1.34	1.30
PFNA	0.85	0.67	0.60	1.30	0.55	0.67	0.94	0.62		
PFDA	0.16	0.13			0.33	0.25				
PFUnDA	0.04	0.03			0.69	0.18	0.21			
PFDoDA	<0.05	<0.05			<0.21	<0.08				
PFBS	<0.02	<0.02			<0.19	0.02				
PFHxS	0.64	0.57	0.50	1.10	0.14	5.00	0.55	1.04	0.35	0.54
PFOS	2.45	1.96	2.00	3.70	2.52	3.80	4.63	3.41	2.07	1.60
FOSA	<0.02	<0.02			<0.1					
MeFOSAA	0.12	0.10								
EtFOSAA	<0.02	<0.02								

Note: FOSA, perfluorooctanesulfonamide; EtFOSAA, N-ethyl perfluorooctane sulfonamido acetic acid; MeFOSAA, N-methyl perfluorooctane sulfonamido acetic acid; PFAS, per- and polyfluoroalkyl substances; PFBS, perfluorobutanesulfonic acid; PFCA, perfluoroalkyl carboxylic acids; PFDA, perfluorodecanoic acid; PFHpA, perfluoroheptanoic acid; PFDoDA, perfluorododecanoic acid; PFHxA, perfluorohexanoic acid; PFHxS, perfluorohexane-1-sulfonic acid; PFNA, perfluorononanoic acid; PFOA, perfluorooctanoic acid; PFOS, perfluorooctanesulfonic acid; PFPeA, perfluoro-n-pentanoic acid; PFSA, perfluoroalkane sulfonic acids; PFUnDA, perfluoroundecanoic acid.

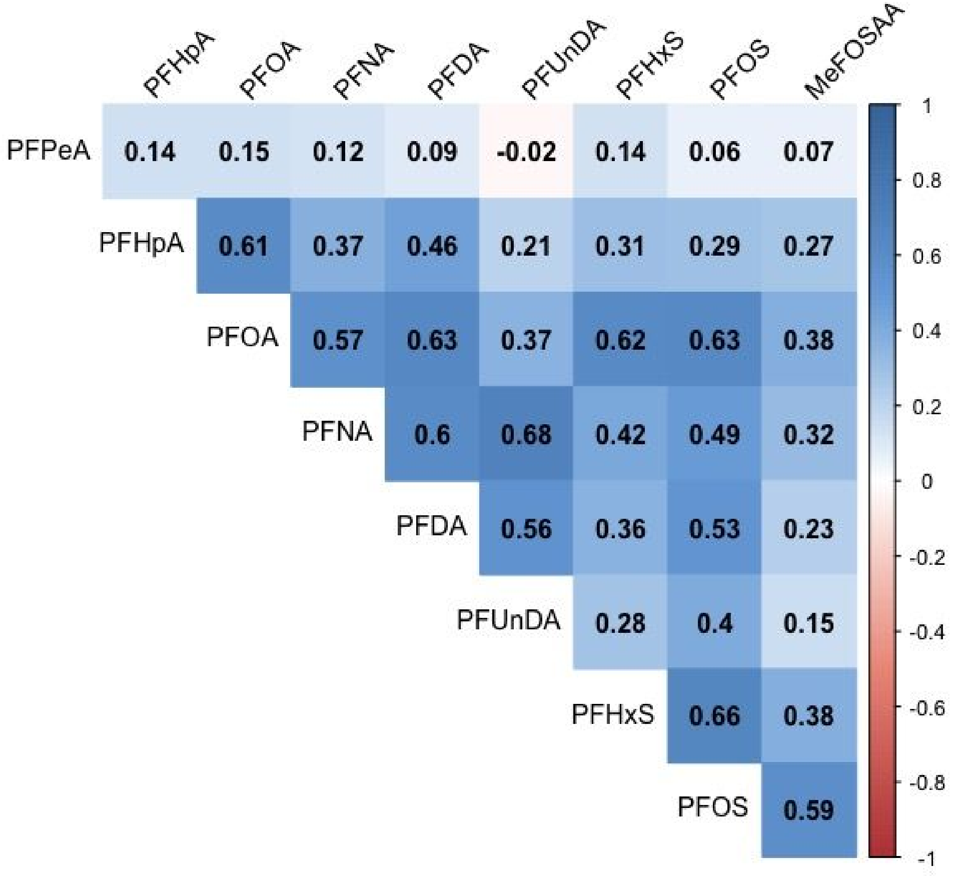

Figure S1. Spearman correlation coefficients among 9 serum PFAS concentrations in 541 CHARGE children. PFAS concentrations below the limit of detection (LOD) were imputed with a value of LOD divided by the square root of 2 prior to computing correlation coefficients. CHARGE, Childhood Autism Risks from Genetics and Environment; MeFOSAA, N-methyl perfluorooctane sulfonamido acetic acid; PFAS, per- and polyfluoroalkyl substances; PFDA, perfluorodecanoic acid; PFHpA, perfluoroheptanoic acid; PFHxS, perfluorohexane-1-sulfonic acid; PFNA, perfluorononanoic acid; PFOA, perfluorooctanoic acid; PFOS, perfluorooctanesulfonic acid; PFPeA, perfluoro-n-pentanoic acid; PFUnDA, perfluoroundecanoic acid.

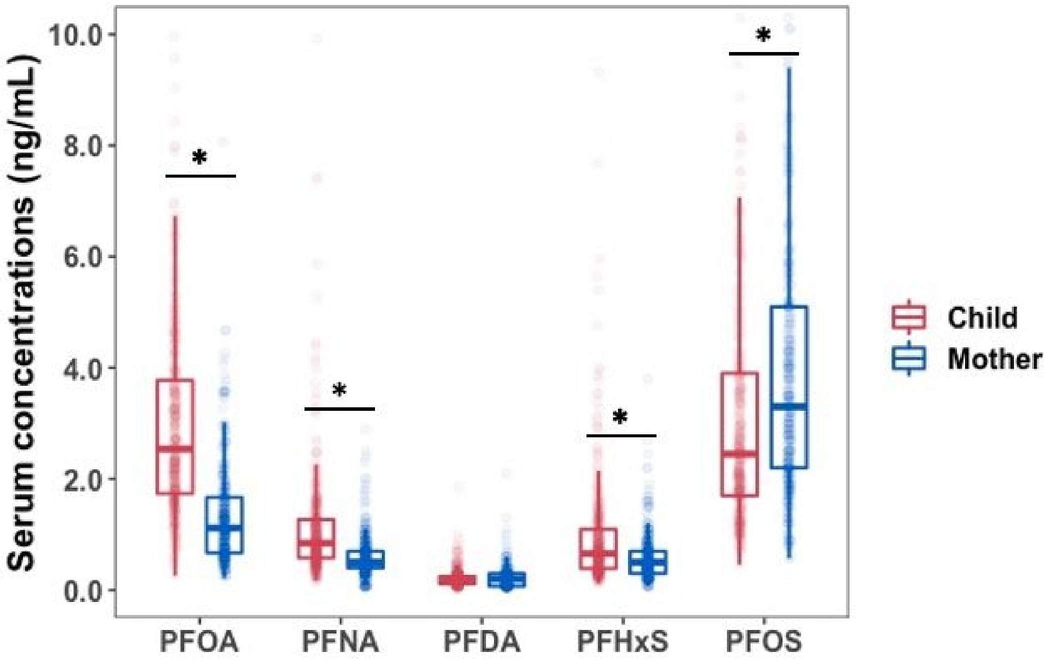

Figure S2. Distributions of maternal and child serum PFAS concentrations in 327 CHARGE mother-child dyads. Horizontal lines in the box represent 25th, 50th, and 75th percentiles from the bottom, and whiskers indicate 5th and 95th percentiles of serum PFAS concentrations. Asterisks represent *p*-value<0.05 from the Wilcoxon rank-sum test comparing child and maternal serum PFAS concentrations. CHARGE, Childhood Autism Risks from Genetics and Environment; PFAS, per- and polyfluoroalkyl substances; PFDA, perfluorodecanoic acid; PFHxS, perfluorohexane-1-sulfonic acid; PFNA, perfluorononanoic acid; PFOA, perfluorooctanoic acid; PFOS, perfluorooctanesulfonic acid.

References

1. Schecter A, Malik-Bass N, Calafat AM, et al. Polyfluoroalkyl compounds in Texas children from birth through 12 years of age. *Environmental health perspectives*. 2012;120(4):590-594.

2. Zhang T, Wu Q, Sun HW, Zhang XZ, Yun SH, Kannan K. Perfluorinated compounds in whole blood samples from infants, children, and adults in China. *Environmental science & technology*. 2010;44(11):4341-4347.

3. Gyllenhammar I, Benskin JP, Sandblom O, et al. Perfluoroalkyl acids (PFAAs) in children's serum and contribution from PFAA-contaminated drinking water. *Environmental science & technology*. 2019;53(19):11447-11457.

4. Papadopoulou E, Sabaredzovic A, Namork E, Nygaard UC, Granum B, Haug LS. Exposure of Norwegian toddlers to perfluoroalkyl substances (PFAS): the association with breastfeeding and maternal PFAS concentrations. *Environment international*. 2016;94:687-694.

5. Ye X, Kato K, Wong L-Y, et al. Per-and polyfluoroalkyl substances in sera from children 3 to 11 years of age participating in the National Health and Nutrition Examination Survey 2013–2014. *International journal of hygiene and environmental health*. 2018;221(1):9-16.

6. Duffek A, Conrad A, Kolossa-Gehring M, et al. Per-and polyfluoroalkyl substances in blood plasma–Results of the German Environmental Survey for children and adolescents 2014–2017 (GerES V). *International Journal of Hygiene and Environmental Health*. 2020;228:113549.
